# Comprehensive analysis of complete chloroplast genome sequence of *Plantago asiatica* L. (Plantaginaceae)

**DOI:** 10.1080/15592324.2022.2163345

**Published:** 2023-01-02

**Authors:** Jing Wu, Jing Zhang, Xiaohu Guo, Nianjun Yu, Daiyin Peng, Shihai Xing

**Affiliations:** a College of Pharmacy, Anhui University of Chinese Medicine, Hefei, China; b Institute of Traditional Chinese Medicine Resources Protection and Development, Anhui Academy of Chinese Medicine, Hefei, China; c MOE-Anhui Joint Collaborative Innovation Center for Quality Improvement of Anhui Genuine Chinese Medicinal Materials, Hefei, China; d Anhui Province Key Laboratory of Research & Development of Chinese Medicine, Hefei, China

**Keywords:** *Plantago asiatica*, plantaginaceae, chloroplast genome, comparative analysis, phylogenetic analysis

## Abstract

*Plantago asiatica* L. is a representative individual species of Plantaginaceae, whose high reputation is owed to its edible and medicinal values. However, the phylogeny and genes of the *P. asiatica* chloroplast have not yet been well described. Here we report the findings of a comprehensive analysis of the *P. asiatica* chloroplast genome. The *P. asiatica* chloroplast genome is 164,992 bp, circular, and has a GC content of 37.98%. The circular genome contains 141 genes, including 8 rRNAs, 38 tRNAs, and 95 protein-coding genes. Seventy-two simple sequence repeats are detected. Comparative chloroplast genome analysis of six related species suggests that a higher similarity exists in the coding region than the non-coding region, and differences in the degree of preservation is smaller between *P. asiatica* and *Plantago depressa* than among others. Our phylogenetic analysis illustrates *P. asiatica* has a relatively close relationship with *P. depressa*, which was also divided into different clades with *Plantago ovata* and *Plantago lagopus* in the genus *Plantago*. This analysis of the *P. asiatica* chloroplast genome contributes to an improved deeply understanding of the evolutionary relationships among Plantaginaceae.

*Plantago asiatica* Linaeus 1753. is an annual or biennial food and herb belonging to the genus *Plantago* of Plantaginaceae^1^ (Figure 1a-1b). The leaves of *P. asiatica* are well-known as vegetables and tea in China or even East Asia.^2^ *P. asiatica* as well as *Plantago depressa* Willd. (Figure 1c) are traditionally used as Plantaginis herb, while their dry seeds are known as Plantaginis semen.^3^ *P. asiatica* is easily cultivated, providing rich resources at low prices. It possesses good prospects in diseases prevention and treatment. Also, modern pharmacological studies have shown that the Plantaginis herb and Plantaginis semen have cholesterol degradation, anti-inflammatory, and anti-oxidation activities and can be used to treat liver disease and obesity.^4–7^ In particular, *P. asiatica* seeds extract rich in phenylethanoid glycosides, flavonoids, iridoids and alkaloids have been reported to prevent heart diseases related to cardiac hypertrophy.^8^ Polysaccharides from *P. asiatica* can be exploited as functional foods attributed to their thermal, antioxidant and radical-scavenging characteristics.^9,10^ Cosmetic and health care applications have been proposed for ethanol extracts from *P. asiatica*.^11^ Besides, this plant has strong tolerance to formaldehyde in the air and good formaldehyde removal ability, either.^12^ Scholars have shed light on compounds identification and clinical functions of *P. asiatica*, which will contribute to maximizing its economic value.^13^ To our knowledge, no study has yet linked *P. asiatica* metabolites to genes in the chloroplast genome, which can be cornerstones of digging key genes and high-quality germplasm resource.^14^

**Figure 1.**
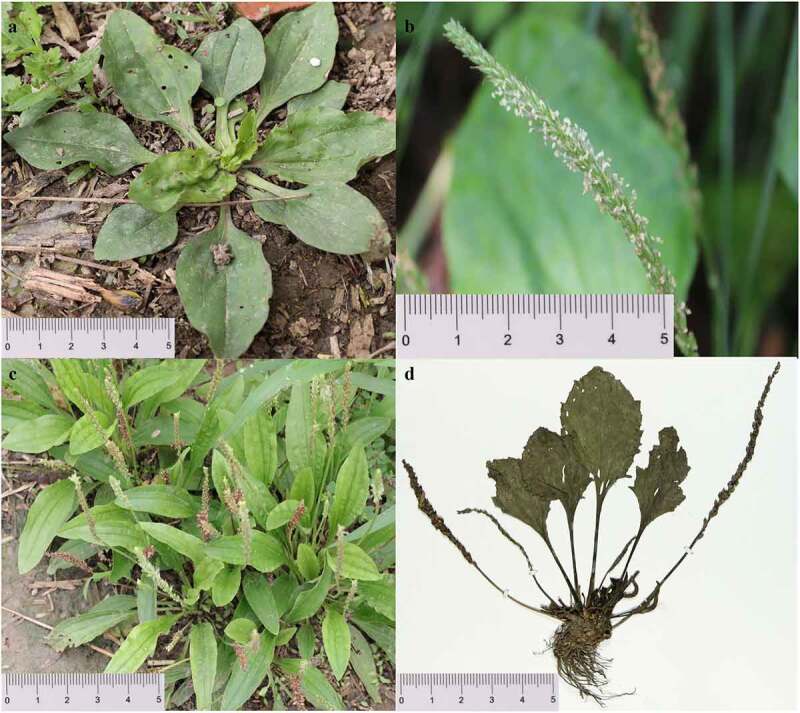

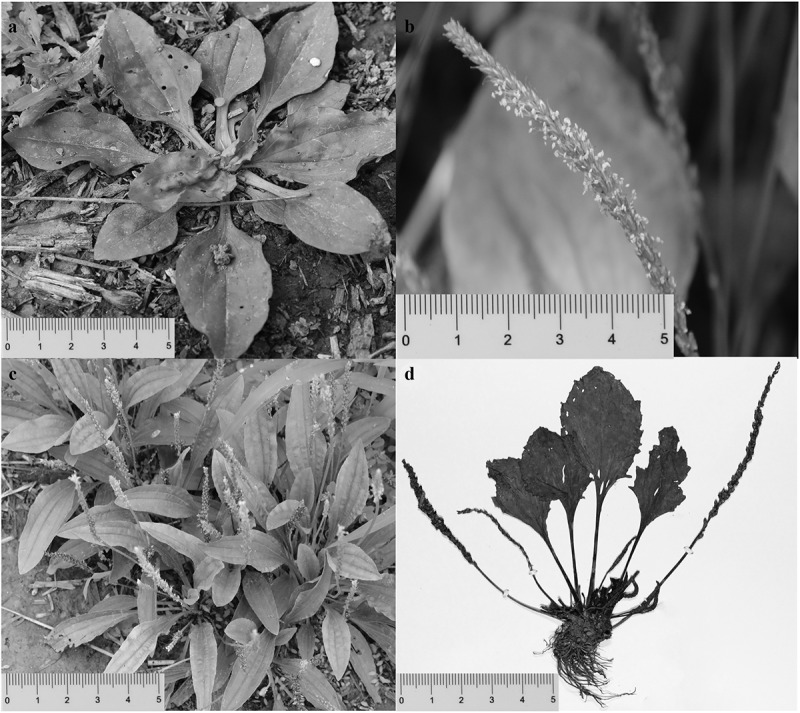
(a). The whole plant of *P. asiatica*. (b). The flowers of *P. asiatica*. (c). The original plant of *P. depressa*. (d). The specimen of *P. asiatica.*

The chloroplast is a key organelle where photosynthesis occurs. Energy in the atmosphere can be converted to starch, pigments, or amino acids here, which are necessary for life activities.^15^ Chloroplast has a relatively independent genetic system called the complete chloroplast (Cp) genome. Genome sequencing has incomparable advantages in analyzing the phylogenetic relationship and further genetic diversity or evolution.^16^ It suits plant taxonomic and adaptive evolutionary studies, especially those involved in interspecific identification.^17^ Functions and evolutionary trajectory of different species in the family Plantaginaceae remained controversial had different active components, leading to different pharmacological effects.^18,19^ DNA barcoding in chloroplast is increasingly popular in phylogeny analyses because of its unmatched accuracy.^20–22^ Moreover, genes detected in the chloroplast can help determine phylogenetic relationships of species belonging to the genus *Plantago*.^23^ Different types of chloroplast genes have been reported to have diverse effects on metabolite regulation. The gene *ycfs* can modulate tetrapyrrole metabolism and biosynthesis, and overexpression of *accD* can increase fatty acid content in leaves.^24,25^ Based on taxonomic studies, Plantaginaceae was regarded as one of the clades of Scrophulariaceae, while it is now formally called Plantaginaceae, although it has drastically expanded from what systematists considered it to be just a few years ago.^26^ An ambiguous perspective always existed that whether Plantaginaceae was more closely relative to Plumbaginaceae in Caryophyllales or Scrophulariaceae in Lamiales.^26–29^ Regrettably, little available chloroplast data was published on *P. asiatica*, the most representative species of Plantaginaceae. Such data could help compare similar species and describe their phylogenetic relationships.

There is a need for better Cp genome data for *P. asiatica*. We use Illumina technology to sequence the whole chloroplast genome of *P. asiatica*, reporting the overall assembly, annotation, and analysis and making comparisons to other species. It will firstly provide a comprehensive analysis that is favorable for the further development and utilization of the resources.

## Results and discussions

### *Primary characteristics of the* plantago asiatica *chloroplast genome*

Clean data of the Cp genome of *P. asiatica* from Illumina sequencing was obtained with 30,787,584 reads representing 4,618,137,600 bp. After optimizing the raw data, the effective ratio of Q20 and Q30 were 97.63% and 93.39%, respectively. The sequencing quality was considered good.

The complete Cp genome of *P. asiatica* has a typical circular shape, the same as most angiosperms, with a length of 164,992 bp, which consists of four distinct regions: a large single-copy (LSC) region of 82,983 bp, a small single-copy (SSC) region of 4,715 bp, and a pair of inverted repeats (IR) regions of 38, 647 bp (Table 1). The GC content can also reflect the characteristics of chloroplast genome composition. It was detected that the GC content of *P. asiatica* Cp genome was 37.98%, which was far lower than the AT content (62.02%). The characteristics of extreme AT richness always existed in chloroplast genomes,^30^ consistent with the consequence of this study. The IR region sequence contains eight genes encoding rRNA, so the corresponding GC content (39.90%) is significantly higher than that of the LSC (36.63%) and SSC (30.27%) regions. The skewness of it was reported to be an instruction of DNA leading and lagging chains, replication starting, and terminal points.^31^

**Table 1.** The primary characteristics of the *P. asiatica* chloroplast genome.

Sequence Region		*P. asiatica*
Total chloroplast genome size (bp)		164,992
LSC length (bp)		82,983
SSC length (bp)		4,715
IR length (bp)		38, 647
Total number of genes		141
Protein-coding genes		95
tRNA genes		8
rRNA genes		38
Genes duplicated by IR		26
Genes with introns		18
GC content	Total (%)	37.98
	LSC (%)	36.63
	SSC (%)	30.27
	IR (%)	39.90
	All genes (%)	38.13

### Gene classification

**Figure 2.**
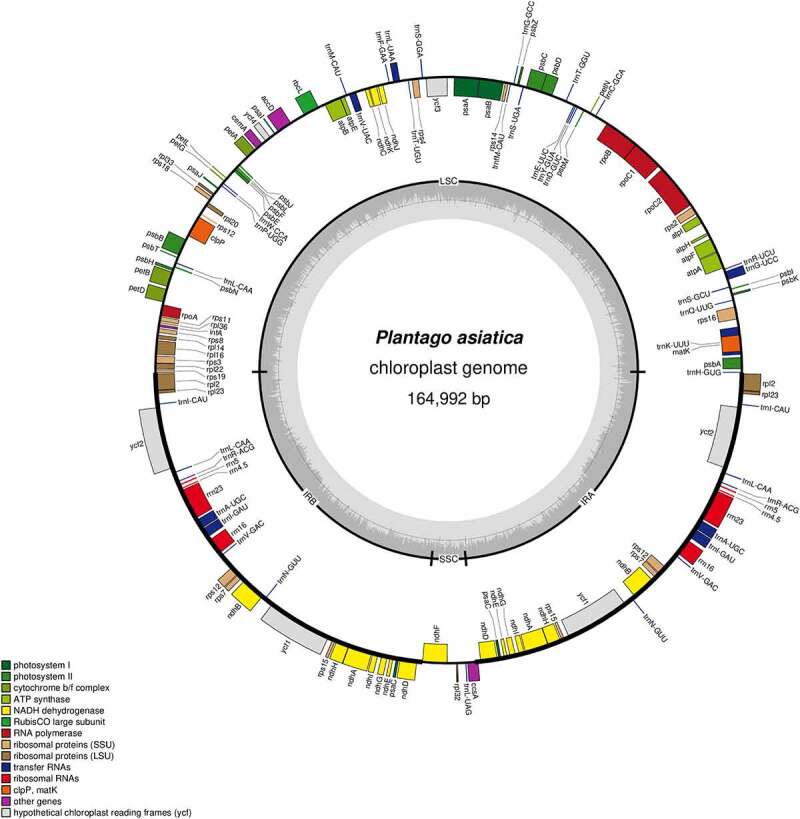

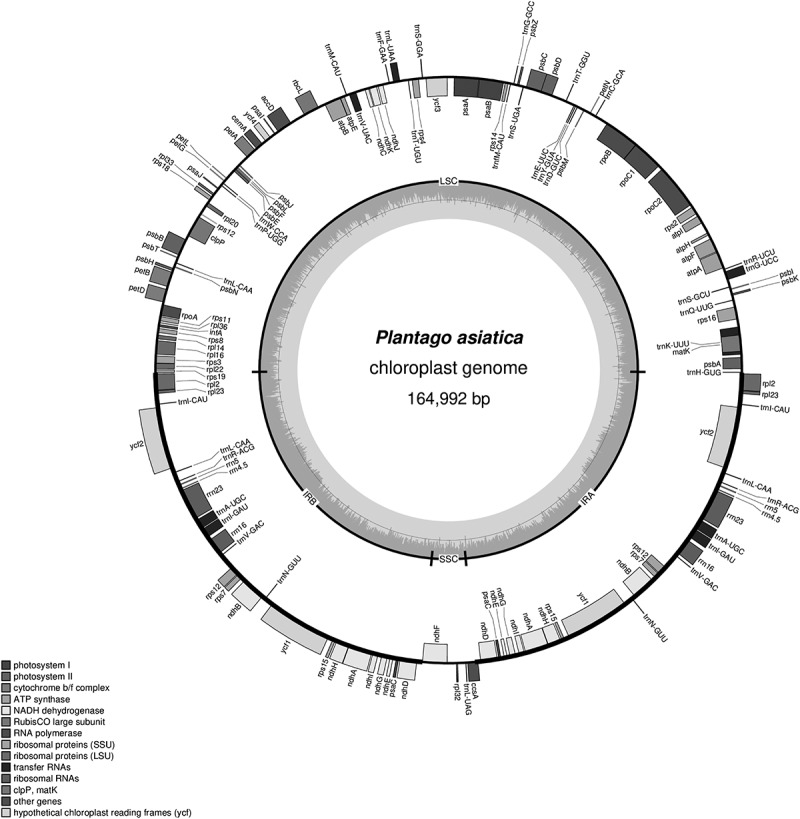
Circular map of the chloroplast genome of *P. asiatica*. Genes drawn inside the circle are transcribed clockwise, while those outsides are transcribed counter-clockwise. Genes are color-coded to imply functional groups. The dark gray area in the inner circle corresponds to the GC content while the light gray corresponds to the AT content of the genome. The SCC, LSC, IRa and IRb regions are noted in the inner circle.

The chloroplast genome encodes 141 genes belonging to three categories, including 95 protein coding genes, 8 ribosomal RNA (rRNA) genes, and 38 transfer RNA (tRNA) genes (Figure 2). When calculating duplicated genes in the IR region, among them, 26 genes were both in the IRa and IRb regions, including 15 protein-coding genes *(ccsA, ndhA, ndhB, ndhD, ndhE, ndhG, ndhH, ndhl, psaC, rpl2, rpl23, rps12, rps15, rps7, ycf2, ycf1*), four rRNA genes (*rrn16, rrn23, rrn4.5, rrn5*) and seven tRNA genes (*trnA-UGC, trnL-CAA, trnl-GAU, trnM-CAU/trnl-CAU, trnN-GUU, trnR-ACG, trnV-GAC*). The SSC region consisted of three protein-coding genes (*ccsA, rpl32, ndhF*) and one tRNA gene (*trnL-UAG*). The LSC region comprised 62 protein-coding genes and 22 tRNA genes (Supplementary Table S1).

Most of the protein-coding genes in the chloroplast genome of *P. asiatica* consisted of one exon. A total of 15 genes (rpoC1*, rps16*, rpl2*, rpl6*, ndhA*, ndhB*, atpF*, petB*, petD*, trnA-UGC*, trnG-UCC*, trnI-GAU*, trnK-UUU*, trnL-UAA*, trnV-UAC*) contained one intron and three genes (clpP1*, ycf3*, rps12*) embodied two introns (Table 2). In the anti-clockwise direction, the region between LSC and SSC is defined as IRa, and the region from SSC to LSC is IRb. What made the difference was the expression model of the trans-spliced gene *rps12* observed due to separate transcription. This was verified as a gene in *P. asiatica* consisting of three exons, whose first exon was located in the LSC region, far from the other two exons distributed to the IR regions. The *rps12* gene ligation between exon 1 and 2 has been confirmed through complementary DNA sequencing of *rps12* mRNA.^32^ Almost all protein-coding genes started with the standard initiator codon ATG, with some exceptions. The *rpoC2* and *rps19* began with GTC and GTG, respectively) which has been reported in the chloroplast genome of other plants.^33^

**Table 2.** The length of introns and exons in genes with introns in *P. asiatica.*

Gene	Region	Exon I (bp)	Intron I (bp)	Exon II (bp)	Intron II (bp)	Exon III (bp)
*atpF*	LSC	145	733	416		
*clpP1*	LSC	69	732	291	640	237
ndhA*	IRA; IRB	551	1074	532		
ndhB*	IRA; IRB	723	677	756		
*ycf3*	LSC	124	708	228	729	155
*petB*	LSC	6	730	642		
*petD*	LSC	8	712	475		
*rpl16*	LSC	9	849	399		
rpl2*	IRA; IRB	391	677	434		
*rpoC1*	LSC	451	748	1613		
*rps12#*	IRA; IRB; LSC	113		228	536	25
*rps16*	LSC	227	136,163	40		
trnA-UGC*	IRA; IRB	38	818	35		
*trnG-UCC*	LSC	23	700	48		
trnI-GAU*	IRA; IRB	37	812	35		
*trnK-UUU*	LSC	37	2444	35		
*trnL-UAA*	LSC	35	513	50		
*trnV-UAC*	LSC	36	579	37		

*Identical genes are duplicated in the IR region with intros. ^#^The *rps12* is a trans-spliced gene with the 5′end located in the LSC region and duplicated in the 3′ end in the IR regions.

Most chloroplast genes may play an essential role in photosynthetic pathways and self-replication. Also, some genes possess special or undefined functions. Based on the functions these genes may perform, genes in the complete Cp genome of *P. asiatica* were sorted into the following types overall: (1) RNA genes; (2) Transcription and translation-related genes; (3) Photosynthesis related genes; (4) Other genes; and (5) Genes of unknown function (Table 3).

**Table 3.** A list of genes found in the plastid genome of *P. asiatica.*

Category for Genes	Group of Gene	Name of Gene				
RNA genes	ribosomal RNA	*rrn5*	*rrn4.5*	*rrn16*	*rrn23*	
	transfer RNA	*trnA-UGC^a^*	*trnC-GCA*	*trnD-GUC*	*trnE-UUC*	*trnF-GAA*
		*trnfM-CAU*	*trnG-GCC*	*trnG-UCC^a^*	*trnH-GUG*	*trnI-CAU*
		*trnI-GAU^a^*	*trnK-UUU^a^*	*trnL-CAA*	*trnL-UAA^a^*	*trnL-UAG*
		*trnM-CAU*	*trnN-GUU*	*trnP-UGG*	*trnQ-UUG*	*trnR-ACG*
		*trnR-UCU*	*trnS-GCU*	*trnS-GGA*	*trnS-UGA*	*trnT-GGU*
		*trnT-UGU*	*trnV-GAC*	*trnV-UAC^a^*	*trnW-CCA*	*trnY-GUA*
Transcription and translation related genes	transcription	*rpoA*	*rpoB*	*rpoC1^a^*	*rpoC2*	
	Small subunit of ribosome	*rps2*	*rps3*	*rps4*	*rps7*	*rps8*
		*rps11*	*rps12^a^*	*rps14*	*rps15*	*rps16^a^*
		*rps18*	*rps19*			
	Large subunit of ribosome	*rpl2^a^*	*rpl14*	*rpl20*	*rpl22*	*rpl23*
		*rpl32*	*rpl33*	*rpl36*	*rpl16^a^*	
Photosynthesis related genes	Rubisco	*rbcL*				
	Photosystem I	*psaA*	*psaB*	*psaC*	*psaI*	*psaJ*
	Photosystem II	*psbA*	*psbB*	*psbC*	*psbD*	*psbE*
		*psbF*	*psbH*	*psbI*	*psbJ*	*psbK*
		*psbL*	*psbM*	*psbT*	*psbZ*	*psbN*
	NADPH dehydrogenase	*ndhA^a^*	*ndhB^a^*	*ndhC*	*ndhD*	*ndhE*
		*ndhG*	*ndhH*	*ndhI*	*ndhJ*	*ndhK*
	ATP synthase	*atpA*	*atpB*	*atpE*	*atpF^a^*	*atpH*
		*atpI*				
	cytochrome b/f complex	*petA*	*petB^a^*	*petD^a^*	*petG*	*petL*
		*petN*				
	Assembly and stability of photosystem I	*ycf3^a^*	*ycf4*			
	cytochrome c synthesis	*ccsA*				
Other genes	Maturase	*matK*				
	translation initiation factor	*infA*				
	carbon metabolism	*cemA*				
	Subunit of acetyl-CoA-carboxylase	*accD*				
	ATP-dependent Clp protease	*clpP1^a^*				
Genes of unknown function	conserved reading frames	*ycf1,*	*ycf2*			

^a^Indicate the intron-containing genes.

### *The gene function annotation of* P. asiatica

The functional annotations from the non-redundant (NR) database of all protein-coding genes were demonstrated (Supplementary Table S2). Among them, 68 protein-coding genes were annotated in species of Plantaginaceae. There were 64 homologous genes to *Plantago media*, while four to *Plantago maritima*. The other group of genes were homologous to those in other families such as *Digitalis lanata, Cajanus cajan*, and *Mucuna pruriens*.

**Figure 3.**
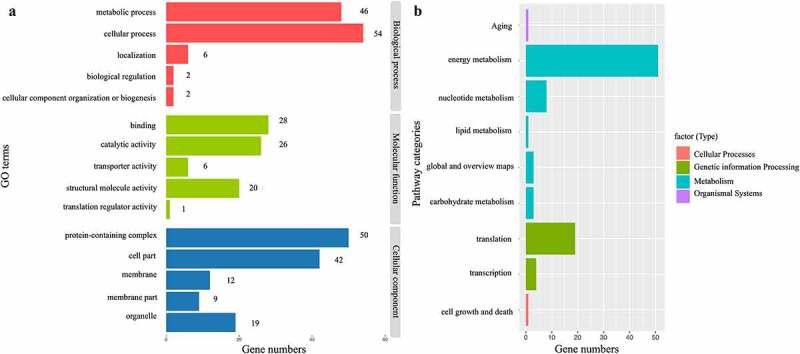

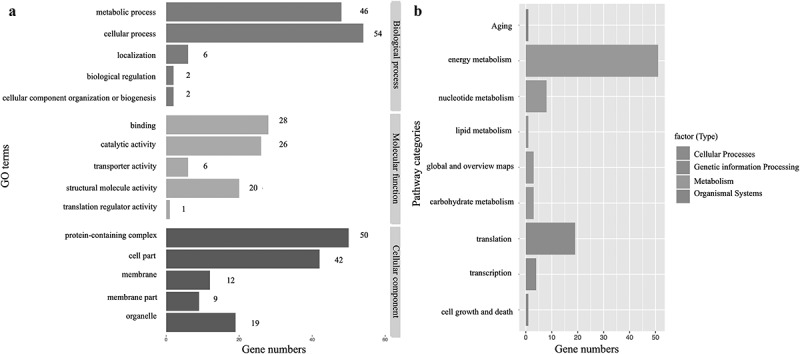
Genes functional annotation in the complete chloroplast genome of *P. asiatica*. (a). The GO classification of genes is based on biological processes, molecular functions, and cellular components. (b). The KEGG pathway categories corresponding to genes in *P. asiatica* chloroplast.

Functional annotation was done using Gene Ontology (GO) terms, including molecular functions (MF), cellular components (CC), and biological processes (BP) subcategories (Supplementary Table S3). The top five GO terms of these three classes are given in Figure 3a. This class was overrepresented among predicted molecular functions, binding, catalytic activity, transporter activity, structural molecule activity, and translation regulator activities. Most genes were related to metabolic and cellular processes attached to BP (57 and 56, respectively), followed closely by protein-containing complex and cell part in CC (52 and 42, respectively). Several genes were also involved in localization and biological regulation, among other functions.

By Kyoto Encyclopedia of Genes and Genomes (KEGG) annotation,^34^ most genes were related to metabolism, especially energy metabolism, nucleotide metabolism, and carbohydrate metabolism (Figure 3b). This was an important aspect of the link between the most dominant term of metabolism in the KEGG pathways and metabolic process in the GO terms. The second group was genetic information processing, such as translation and transcription. Besides, these genes were also characterized and distributed to 15 specific pathways (Supplementary Table S4). Thirty-two genes were predicted to participate in the most recurrent term “Photosynthesis”, mainly in the chloroplast, where various metabolites were produced. Notably, the gene *accD* in the chloroplast of *P. asiatica* was related to fatty acid biosynthesis and metabolism, which had been verified to improve fatty acid content in tobacco, extend leaf longevity, and result in a two-fold increase in seed yield.^24^ The *clpP1* gene encoded serine protease, which ensures normal physiological metabolic processes by degrading and removing misfolded proteins.^35^ The *clpP* disruption also induced will lead to deformed leaf development and affect the accumulation of metabolites.^36^ Hence, further work on exploring the functions of genes in chloroplast about metabolites is of great significance in *P. asiatica*.

### SSRs and long repeat analysis

**Figure 4.**
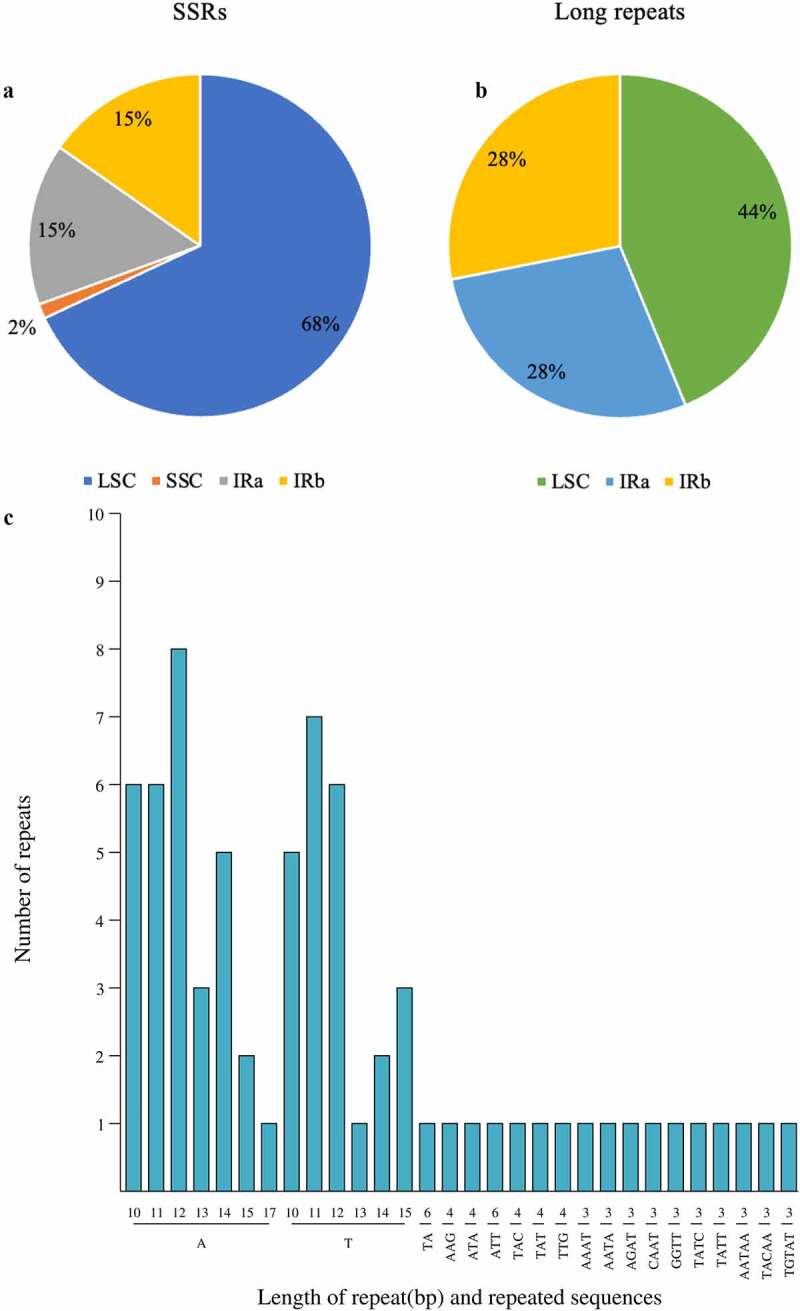

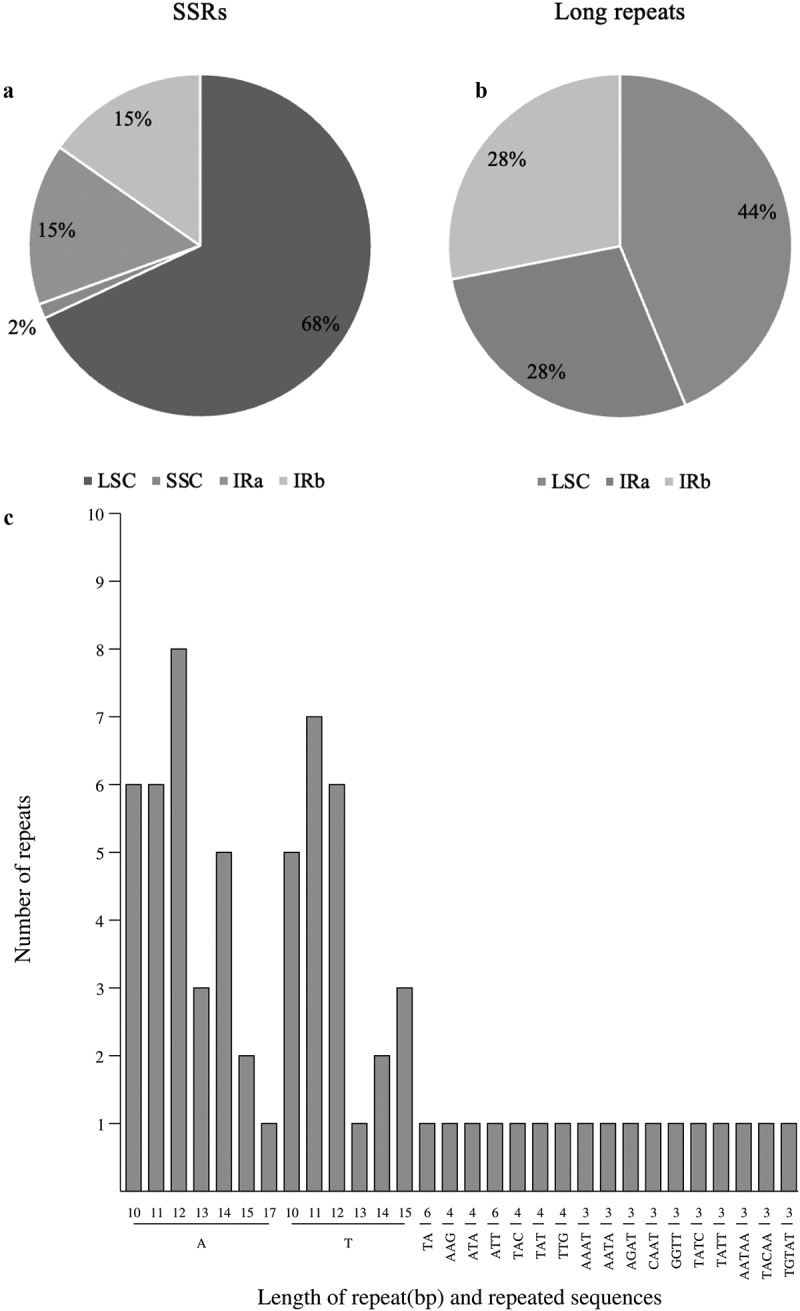
The type, number, and presence of SSRs and long repeats in the Cp genome of *P. asiatica*. (a) Presence of SSRs in the LSC, SSC, IRa, and IRb regions. (b) Presence of long repeats in the LSC, IRa, and IRb regions. (c) Type and number of SSRs in the Cp genome of *P. asiatica.*

Plant genome SSRs are well-described as microsatellites and short tandem repeats (STRs). They were widely employed for molecular genetic markers and plant typing.^37^ A total of 72 short repeats were detected, mostly single nucleotide repeats and mainly A (51) and T (56) (Supplementary Table S5). The bulk of SSRs was located in the LSC region (n = 49), with 1, 11, and 11 SSRs in the SSC, IRa, and IRb regions, respectively (Figure 4a). Of these, 30 short repeat types were diverse from each other, which were comprised of five SSR types: mono-nucleotide (n = 13), dinucleotide (n = 1), trinucleotide (n = 6), tetranucleotide (n = 7), and pentanucleotide (n = 3) (Figure 4c). Among these SSRs, 21 SSRs were only composed of A or T bases. In the other nine simple repeats, more than half of the sequence consisted of A or T bases but rarely covered tandem G or C bases. Therefore, SSRs in the *P. asiatica* Cp chloroplast genome were AT-rich, following the overall AT abundance of the complete sequence of the chloroplast. This finding had been reported in other chloroplast genomes, with speculation that it may be due to A-T transformation being easier than G-C transformation.^38^ The SSRs unevenly distributed in the *P. asiatica* chloroplast genome can provide a theoretical basis for developing SSR molecular markers. Moreover, we also analyzed the large repeat sequence in the chloroplast. A total of 49 long tandem repeats were identified in the *P. asiatica* chloroplast genome (Supplementary Table S6), with the largest percentage of 44% being distributed to the LSC region. There was no long repeat located in the SSC region, while 28% repeat sequences in IRa and IRb regions (Figure 4b). Long tandem repeats, unlike SSRs, were dispersed in the whole genome, with 27 forward (F) repeats and 22 palindromic (P) repeats. The long repeats ranged from 40 to 659 bp.

### Codon usage bias

**Figure 5.**
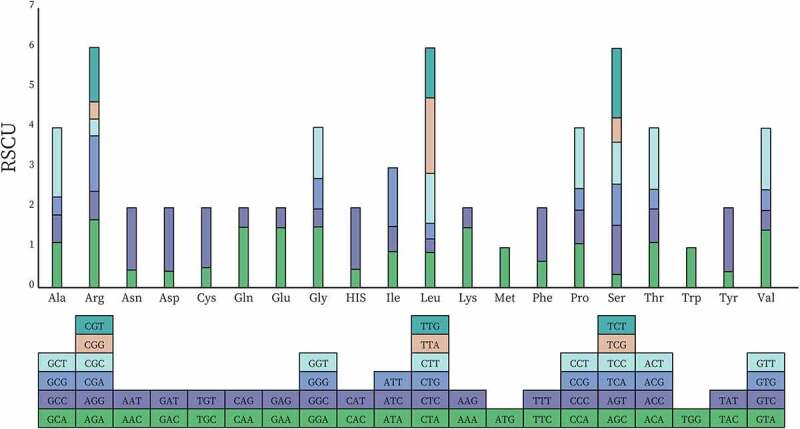

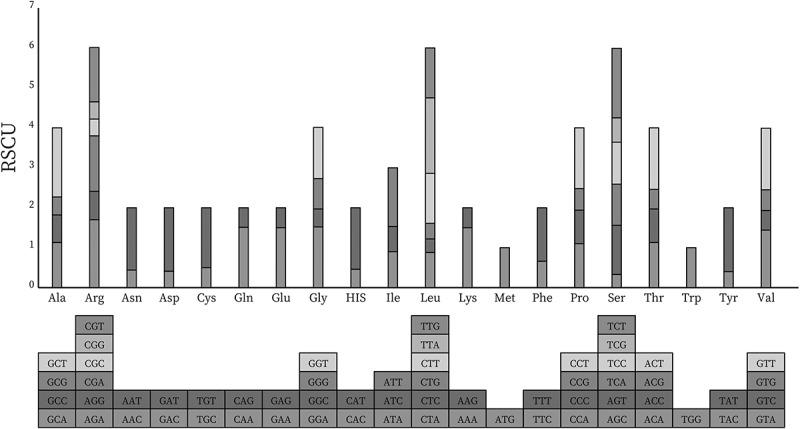
RSCU histogram of *P. asiatica*. The blocks underneath stand for different codon encoding amino acids. The columns on the top depict the sums of RSCU values of the 20 amino acids.

Codon usage exerts a significant influence on shaping genome evolution. As one of the numerous factors that can decide codon usage, mutational bias has a particularly crucial role in shaping the plastome evolutionary phenomenon.^39^ These factors have been demonstrated to influence codon usage at the mutational and translational levels, but mutational pressure is the dominant force at the level of chloroplast genomes. Other mechanisms that potentially help shape the codon use bias include strand asymmetry, which causes strand-specific bias in organelle genomes.^40^ Because of codon degeneracy, each amino acid has one to six codons. Above all, understanding the mutational and translational bias of codon usage can help explore chloroplast evolution. The inequality of synonymous codon usage results from natural selection, mutation, and genetic drift. Relative synonymous codon usage (RSCU) and codon usage measures in *P. asiatica* such as Nc (number of codons) values suggesting the degree of the codon usage bias, frequency of A, T, G, and C were exhibited (Supplementary Table S7). The Nc value for each protein-coding gene ranged from 23.64 (*psbI*) to 58.78 (*clpP1*), among which Nc values for *rps16, rpl36*, and *psbT* could not be calculated because they contained no amino acids with synonymous codons. Consequently, there were 28,949 codons detected in all genes (Figure 5, Supplementary Table S8). The leucine encoded by six types of codons was the most abundant amino acid in this chloroplast genome (3,220 codons, 11.12%). The second abundant was isoleucine (2,362 codons, 8.16%). However, cysteine only accounted for 1.22% of amino acids. The highest value of RSCU was UUA (1.82), and the lowest was AGC (0.315). The RSCU value of 32 codons was greater than 1.00, of which 28 codon bases ended in A or U, and the remaining four ended in G. Similar to *Albizia julibrissin*,^41^ the G or C terminal codons were used more frequently than expected (RSCU value < 1). And in *P. asiatica*, the stop codons preferred the use of TAA.

### Prediction of RNA editing sites

RNA editing in the plastid can contribute to regulating chloroplast development.^42^ We predicted the 47 editing sites in 16 genes (Table 4), all of which were C to T conversions. These changes in RNA editing sites all led to the transformation of amino acids, including TCA (S) to TTA (L), GCG (A) to GTG (V), CAT (H) to TAT (Y), ACC (T) to ATC (I), TCG (S) to TTG (L), and so on. The most common change was serine (S) to leucine (L), accounting for 19 (40.42%). Amino acid conversion from hydrophilic to hydrophobic can lead to an increase in protein hydrophobicity.^43^ RNA editing enriches the genetic information of the genome as a single gene translate to different proteins. Some genes in the chloroplast must be edited to be translated normally. The gene *rpl2* in maize chloroplast can be transcribed and translated normally only after the initiation codon ACG mutating into ATG.^44^ In our study, the *ndhB* gene had the largest number of editing sites, up to 16. The next gene, *rpoC2*, has six editing sites, followed by *rpoB* (n = 5), *rpl20* (n = 4), *matK* (n = 3), *ndhA* (n = 3), *ndhD* (n = 3), *rps14* (n = 3), *rpoA* (n = 2). Only one editing site was predicted for genes *accD, atpA, ccsA, ndhG, psbE, rpl2*, and *rpoC1*, respectively.

**Table 4.** The predicted RNA editing sites in *P. asiatica* chloroplast genome.

Gene	Codon Position	Amino Acid Position	Codon (Amino Acid) Conversion	Score
*accD*	1090	364	CCA (P) = > TCA (S)	1
*atpA*	791	264	CCC (P) = > CTC (L)	1
*ccsA*	227	76	ACC (T) = > ATC (I)	0.86
*matK*	1001	334	GCC (A) = > GTC (V)	0.86
	1154	385	ACC (T) = > ATC (I)	1
	1465	489	CAT (H) = > TAT (Y)	0.86
*ndhA*	341	114	TCA (S) = > TTA (L)	1
	566	189	TCA (S) = > TTA (L)	1
	1064	355	TCC (S) = > TTC (F)	1
*ndhB*	95	32	TCA (S) = > TTA (L)	1
	413	138	CCA (P) = > CTA (L)	1
	532	178	CAT (H) = > TAT (Y)	1
	557	186	TCG (S) = > TTG (L)	0.8
	692	231	TCT (S) = > TTT (F)	1
	776	259	TCA (S) = > TTA (L)	1
	782	261	TCA (S) = > TTA (L)	1
	857	286	ACT (T) = > ATT (I)	1
	869	290	ACA (T) = > ATA (I)	1
	1238	413	TCC (S) = > TTC (F)	1
	1427	476	CCA (P) = > CTA (L)	1
*ndhD*	29	10	ACG (T) = > ATG (M)	1
	94	32	CTT (L) = > TTT (F)	1
	905	302	TCA (S) = > TTA (L)	1
*ndhG*	320	107	ACA (T) = > ATA (I)	0.8
*psbE*	214	72	CCT (P) = > TCT (S)	1
*rpl2*	593	198	GCG (A) = > GTG (V)	0.86
*rpl20*	59	20	TCT (S) = > TTT (F)	1
	221	74	GCA (A) = > GTA (V)	0.86
	302	101	TCA (S) = > TTA (L)	0.86
	328	110	CCG (P) = > TCG (S)	0.86
*rpoA*	82	28	CAT (H) = > TAT (Y)	0.86
	329	110	GCC (A) = > GTC (V)	0.86
*rpoB*	211	71	CAT (H) = > TAT (Y)	1
	473	158	TCA (S) = > TTA (L)	0.86
	551	184	TCA (S) = > TTA (L)	1
	566	189	TCG (S) = > TTG (L)	1
	2426	809	TCA (S) = > TTA (L)	0.86
*rpoC1*	305	102	GCG (A) = > GTG (V)	1
*rpoC2*	832	278	CTT (L) = > TTT (F)	0.86
	2293	765	CGG (R) = > TGG (W)	1
	2324	775	GCT (A) = > GTT (V)	1
	2629	877	CCC (P) = > TCC (S)	0.86
	3352	1118	CAC (H) = > TAC (Y)	0.86
	3713	1238	TCG (S) = > TTG (L)	0.86
*rps14*	80	27	TCA (S) = > TTA (L)	1
	149	50	CCA (P) = > CTA (L)	1
	190	64	CTT (L) = > TTT (F)	0.86

### Comparative analysis of the chloroplast genomes

**Figure 6.**
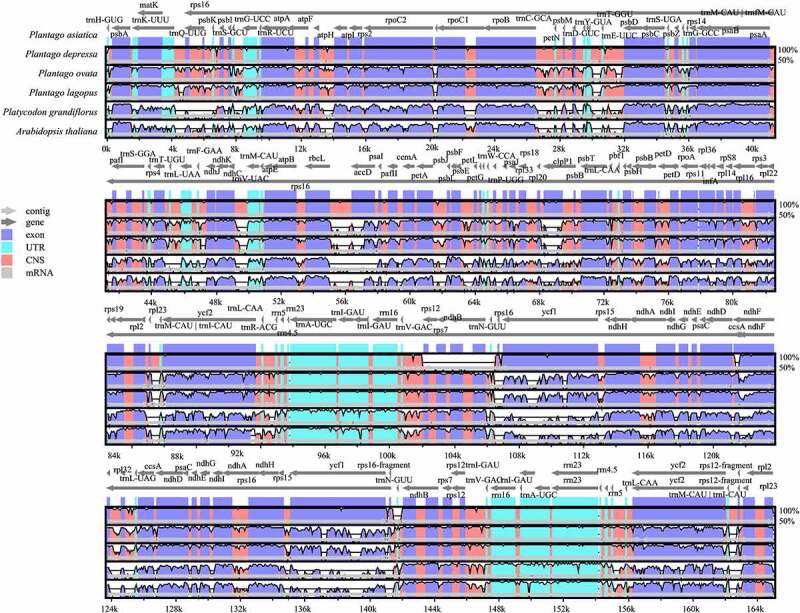

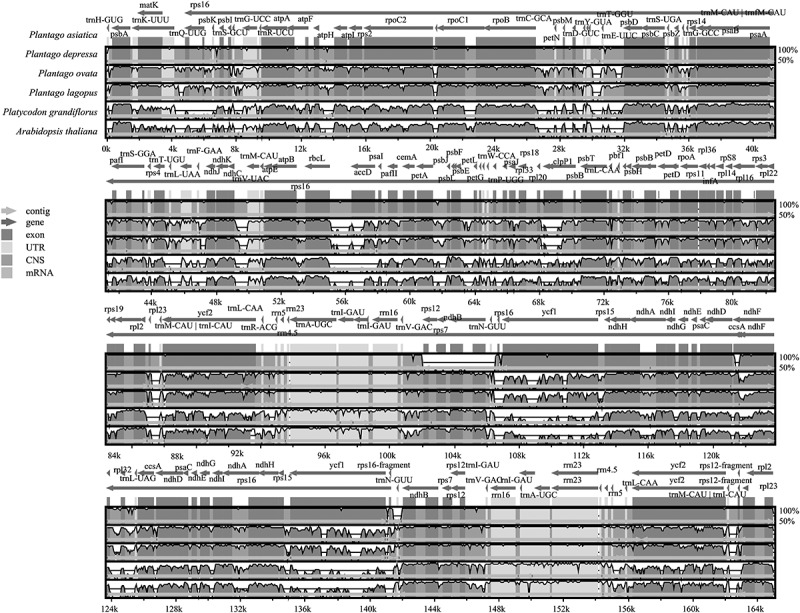
Comparison of the Cp genome sequences of *P. asiatica, P. depressa, P. ovata, P. lagopus, P. grandiflorus*, and *A. thaliana* generated using mVISTA. Gray arrows symbolize the position and direction of the genes. Red and blue areas indicate intergenic and genic regions, respectively. Black lines represent regions of sequence identity with *P. asiatica*, with a 50% identity cutoff. Dashed rectangles denote highly divergent regions when *P. asiatica* compared to *P. depressa, P. ovata, P. lagopus, P. grandifloras*, and *A. thaliana.*

To explore the potential sequence divergence of *P. asiatica*, we used annotation of the model plant *Arabidopsis thaliana*, the far relative species *Platycodon grandiflorus* and three species published in the genus *Plantago*. We plotted the total Cp genome identities in mVISTA (Figure 6). Overall, sequence divergence was low across the family Plantaginaceae plastid genomes compared to other species. Among them, *P. depressa* possessed the highest similarity with *P. asiatica*, while the distantly related species *P. grandiflorus* had marked differences when using the *A. thaliana* chloroplast genome as the external reference sequence. It is worth noting that in chloroplasts of species in the same family, Plantaginaceae and other families, the substitution rates in LSC and SSC regions were mildly higher than in the IR regions.^45^ This phenomenon has been described in many plants, which may be because of copy correction by gene conversion or the presence of conserved rRNA genes in the IR region.^46^

As expected, the non-coding regions showed higher sequence divergence than coding regions.^47^ The coding regions of the *clpP1, accD, ndhD, ccsA, ycf1*, and *ycf2* genes were quite diverse among the species in the family Plantaginaceae. The *clpP1* and *ycf1* genes were also different between *P. asiatica and P. depressa*. It is suggested that these conserved coding genes in chloroplast can be used to trace the phylogenetic relationships among lots of eudicot plants,^48^ *P. asiatica* included.

**Figure 7.**
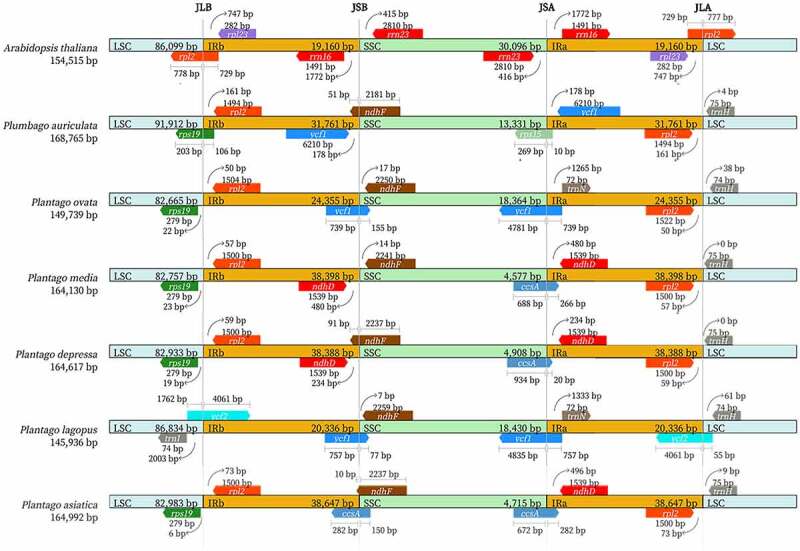

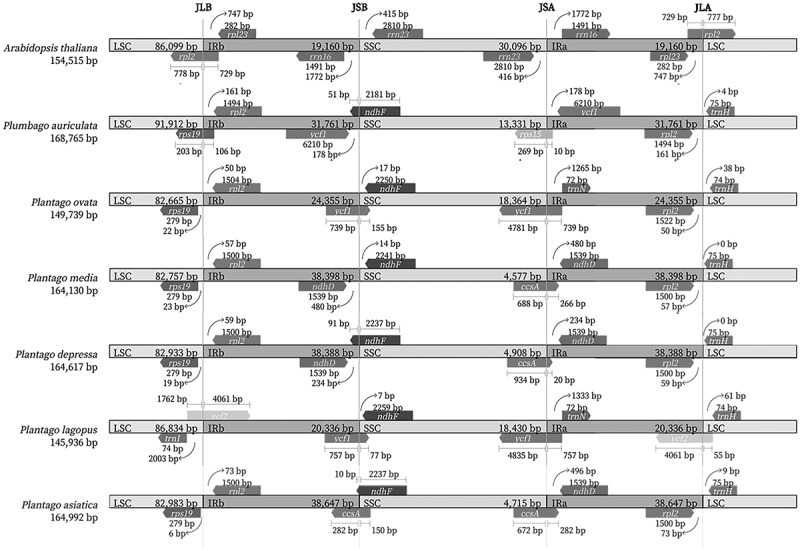
Comparison of the borders of the LSC, SSC, and IR regions of *P. asiatica, P. lagopus, P. depressa, P. media, P. ovata, P. auriculata* and *Arabidopsis thaliana.*

Contraction and expansion in the IR area boundaries are recognized as recurrent evolutionary processes that have led to the observed variance in chloroplast genome size.^49^ The variation of genes situated at plastome termini and boundary shifts (IR-SC) in four junctions (JLB -LSC/IRb, JSB -IRb/SSC, JSA -SSC/IRa, and JLA -IRa/LSC) were investigated. Detailed comparisons of JLB, JSB, JSA, and JLA in *P. asiatica, P. lagopus, P. depressa, P. media, P. ovata*, far relative species *Plumbago auriculata* and *A. thaliana* were depicted in Figure 7. Compared with *A. thaliana*, the *ndhF* gene overlapped at the JSB boundary of *P. asiatica, P. lagopus, P. depressa, P. media, P. ovata, P. auriculata*, while this gene was not in the same position of the *A. thaliana*. The junction line between SSC and IRa intersected the *ycf1* or *ccsA* gene in the family Plantaginaceae, and the gene *rps15* was observed in the JSA of *P. auriculata*. In the family Plantaginaceae, *P. asiatica* showed the largest-scale inversion of expanded IR regions among these 5 species, which may be the main cause of its largest size of Cp genome. The boundaries in four junctions of *P. asiatica* were similar with *P. depressa* and *P. media* but differed from *P. ovata* and *P. lagopus*. There was some degree of extension into the SSC observed in *P. asiatica*, which to some extent occurred in other angiosperms.^50^The gene *rpl2* extended into the IRb regions ranging from 50 bp to 73 bp except *P. lagopus*, whose *rpl2* gene was located in LSC region. The same extension can be uncovered in the *ycf1* gene. It traversed the SSC and IRa regions (LR line) of *P. ovata* and *P. lagopus*. Significantly, *P. ovata* and *P. lagopus* had smaller Cp genome size than other species because the *ycf1* gene was partly relocated to the SSC region by IR contraction. Contrastingly, in *P. asiatica* as well as the two similar species, *ycf1* gene did not extend up to the SSC region and was completely duplicated in IRs. The extensions of these genes in *P. asiatica* may be why this was the largest genome among these five species in the family Plantaginaceae, leading to a smaller SSC region as reported in other species.^50^

### Phylogenetic relationship analysis

**Figure 8.**
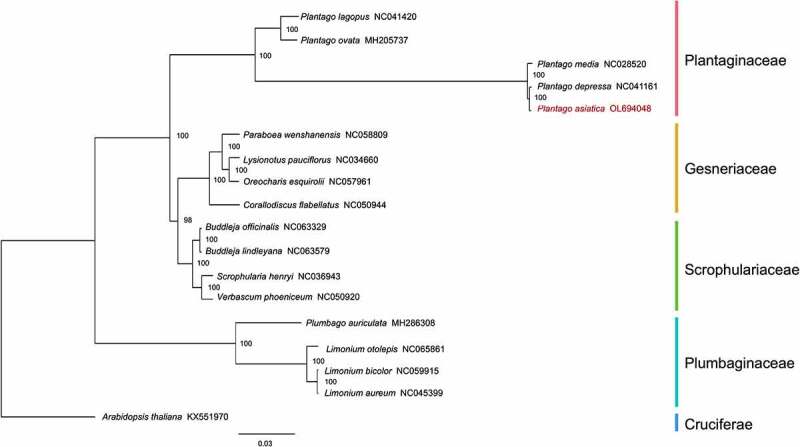

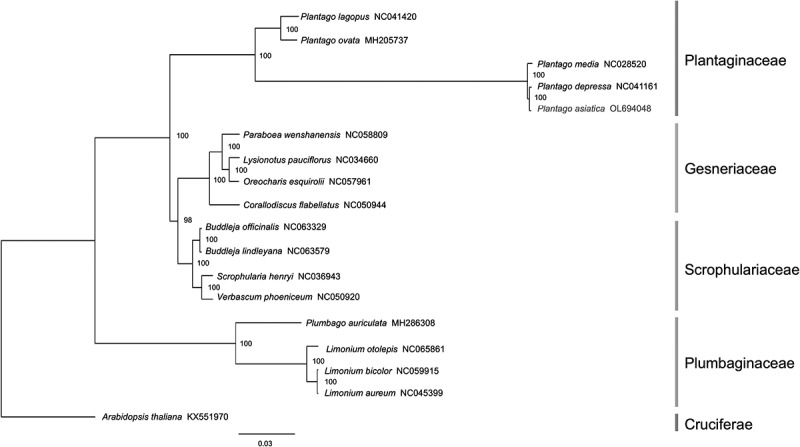
Phylogenetic tree plotting using ML method, based on aligning the completed chloroplast genome sequences of *Plantago asiatica* L. and 17 other representative species. The numbers above the nodes are support values with ML bootstrap values. The distance bar was implied by the ML method.

Phylogenetic relationship analysis in the light of chloroplast genome is more convenient than nuclear genome in tracing plant species lineages and species identification.^48^ To resolve the phylogenetic position of *P. asiatica* in the genus *Plantago* and determine whether it is closer to Plumbaginaceae in Caryophyllales or Scrophulariaceae in Lamiales, four typical species in Gesneriaceae, Scrophulariaceae, and Plumbaginaceae were chosen, as well as five species in Plantaginaceae. The topology of the trees constructed by two different methods [maximum likelihood (ML) and Bayesian inference (BI)] was identical, confirming the robustness of our data. The bootstraps (BP) in ML analysis were presented (Figure 8). BI phylogram showing branch lengths was included in Supplementary Figure S1. In the phylogenetic tree, the species can be regarded as three clades. Clade I stood for Plumbaginaceae in Caryophyllales and occupied the basal position. It was phylogenetically distant from other families. Clade II was composed of Scrophulariaceae and Gesneriaceae, supported with 98 bootstrap BP values. Plantaginaceae was divided into Clade III, consisting of two subclades with a BP value of 100. Consequently, with strong support, it uncovered that *P. asiatica* is closely related to *P. depressa* (NC041161). *P. asiatica, P. depressa*, and *P. media* (NC028520) were members of one subclade, while *P. ovata* (MH205737) and *P. lagopus* (NC041420) were distributed to the other branch in Plantaginaceae. What followed the conclusion was that *P. asiatica, P. depressa*, and *P. media* are in the subgenera of Plantago, but *P. ovata* and *P. lagopus* are in the *Psyllium* subgenera.^51^ Moreover, compared with species in Gesneriaceae and Scrophulariaceae, *P. asiatica* had the farthest relationship with species in Plumbaginaceae.

**Figure 9.**
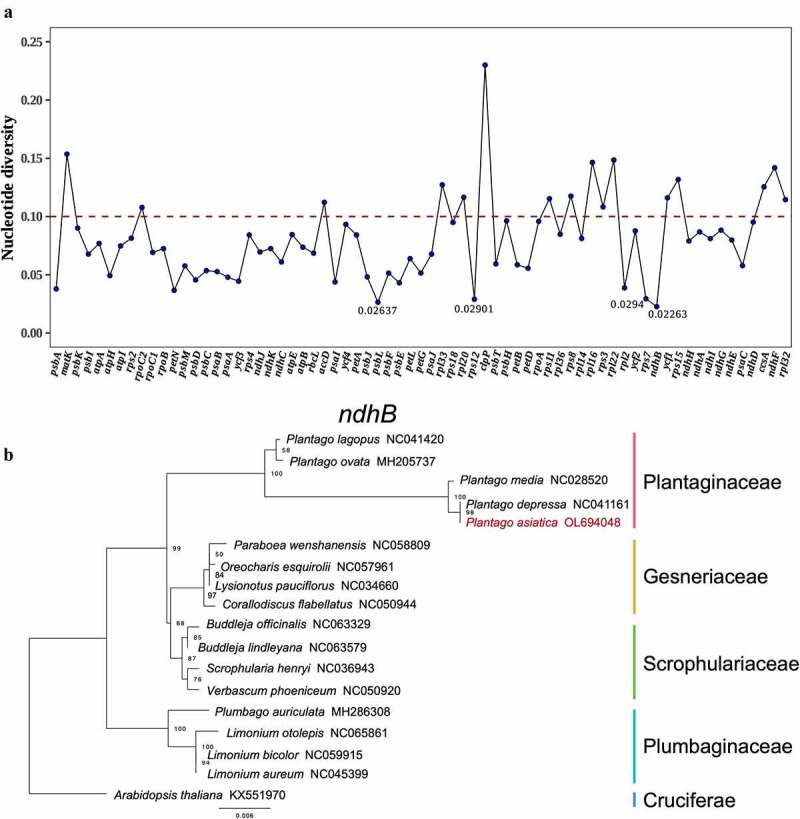

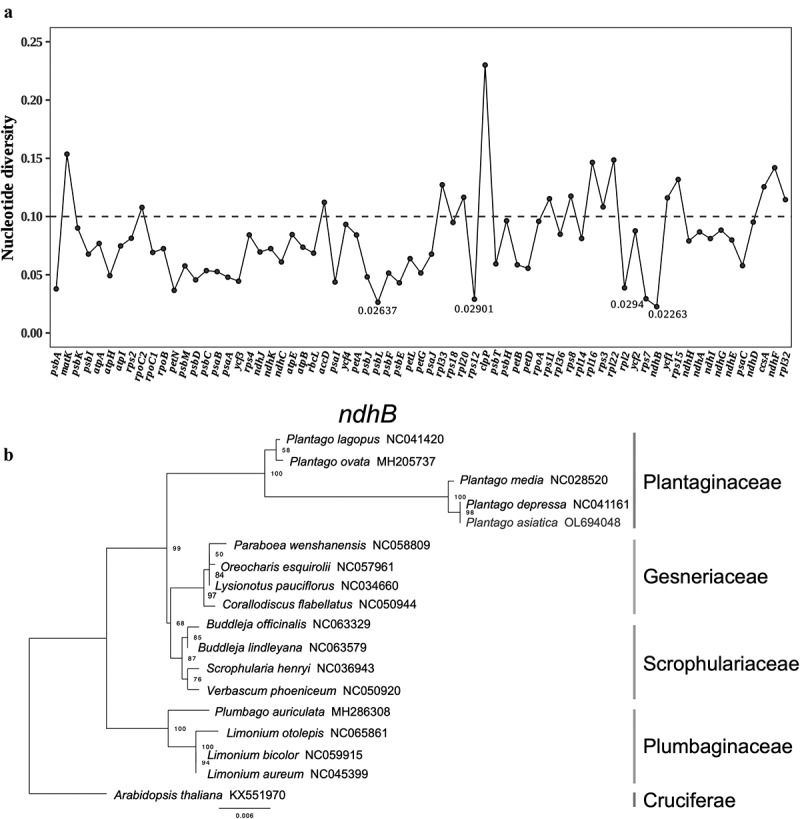
(a). Pi values of the protein coding genes. (b). Phylogenetic tree by maximum likelihood method of *ndhB* gene.

Furthermore, a DNA polymorphism analysis (Pi) was used to detect low variable sites in the chloroplast genome among 18 species, showing a phylogenetic tree of only highly conserved gene sequences (Figure 9a, Supplementary Table S9). The average Pi was 0.08059. Genes with high Pi values can be used as molecular markers for plant identification and phylogenetic analysis.^52^ In particular, the *clpP1* (Pi = 0.23005) and *matK* (Pi = 0.1536) appeared highly diverse among these species. Although the morphological and chloroplast sequence were similar between *P. asiatica* and *P. depressa*, the *clpP1* and *ycf1 genes* were hyper-variable regions between *P. asiatica* and other species. On the basis of the results and the sequence divergence analysis, we proposed that the *clpP1* and *ycf1* genes with comparatively high sequence deviation, are good for interspecies phylogenetic analysis. Moreover, the four genes with the lowest Pi values were chosen to reveal phylogenetic roles: *ndhB* (0.02263), *psbL* (0.02637), *rps12*(0.02901), and *rps7*(0.0294). Using BI and ML methods, phylogenetic analysis was also performed with these four highly conserved genes. The ML and BI phylogenetic trees of the four genes are shown in Supplementary Figures S2–S5. The most highly conserved *ndhB* gene was created with the same topology consistent with the complete genomes, which enabled more inference of the relationship based on the phylogenetic studies (Figure 9b).^53^ The trees generated using BI or ML methods suggested *P. asiatica* and *P. depressa* formed a single clade with high bootstrap (100%) and BI support. In addition, the position of *P. asiatica* and the family Plantaginaceae followed the topologies from previous phylogenetic studies within Lamiales.^53,54^ However, the comprehensive analysis of *P. asiatica* was not operated. And Cp genome from Scrophulariaceae was not included in the previous study. Thus, the present phylogeny verifies that Plantaginaceae is closer to Gesneriaceae and Scrophulariaceae in Lamiales than Plumbaginaceae in Caryophyllales.

## Materials and methods

### Plant materials

Fresh and well-grown leaves of *P. asiatica* were obtained from a single individual from the medicinal botanical garden of Anhui University of Chinese Medicine Anhui, China (N31°56ʹ58”; E117°23ʹ38”), followed by the identification of associate Professor Qingshan Yang from the Anhui University of Chinese Medicine. A voucher specimen of this plant was stored in the Center of Herbarium, Anhui University of Chinese Medicine, Hefei, China (AhtcmH, yxy.ahtcm.edu.cn/info/1006/6713.htm, Shi-hai Xing, xshshihai@163.com, under the voucher number 20211104) (Figure 1d). No ethical approval/permission is required in this study. Because the material used in the study is a so normal plant that can be used as vegetable. It’s neither an endangered or protected plant nor collected in any protected land. Furthermore, there’s no relative local and national regulations or guidelines in China about collecting this plant. The sample was legally collected in accordance with guidelines provided by the authors’ institution and national or international regulations. Field studies was complied with local legislation.

### DNA extraction, filtering of raw reads and genome sequencing

Leaves were snap-frozen with liquid nitrogen prior to being stored at −80°C. Total mixed genomic DNA was extracted by the CTAB method, with minor changes. DNA quality and integrity were confirmed by gel electrophoresis and Nanodrop methods. DNA libraries with different indices were multiplexed and loaded on an Illumina HiSeq instrument according to the manufacturer’s instructions (Illumina, San Diego, CA, USA). Sequencing was carried out using a 2 × 150 paired-end (PE) configuration, and image analysis and base calling were conducted by the HiSeq control software (HCS), OLB, and GAPipeline-1.6 (Illumina) on the HiSeq instrument.

### Chloroplast genome assembly and analysis

The length of the whole chloroplast genome was predicted using KmerGenie.^55^ Short reads were quality controlled and de novo, then assembled using velvet (version 1.2.10), followed by gaps being filled using SSPACE (version 3.0)^56^ and GapFiller (version 1–10).^57,58^ Based on the clean data, the Cp genome of *P. asiatica* was assembled using NOVOPlasty 2.7.2^59^ and auxiliary software Spades^60^ on all the contigs, which utilized the complete Cp sequences of *P. depressa* (GenBank: NC041161) as the reference genome. The assembled Cp genome sequence of *P. asiatica* was submitted to NCBI with GenBank OL694048.

### Chloroplast gene annotation

For elucidation of these genes, an online tool of the Dual Organellar GenoMe Annotator (DOGMA) program^61^ was initially applied to annotate the *P. asiatica* chloroplast genome, whose protein-coding genes identity was set to 50 and hit number to 10. It is worth noting that start and stop sites of these annotated protein-coding genes needed manual correction. Ribosome RNA was detected by aligning rRNA sequences from other chloroplast genomes to ginseng chloroplast genome sequence using BLAST with global coverage and identified ≥ 90%, and tRNA was identified by tRNAscan-SE^62^ with default parameters. A circular Cp genome map was drawn using the OGDRAW program.^63^ Then, each divided sequence was searched against the NR database using BLASTN, GO annotation by Blast2GO, and the KEGG database for identification.

### Analysis of long repeats and SSRs

The web-based REPuter program^64^ (https://bibiserv.cebitec.uni-bielefeld.de/reputer) was employed to visualize the four repetitive sequences of the Cp genome, the forward, reverse, palindrome, and complement sequences included. As for all the repeat types, the constraints set in REPuter contributed to identifying all the 90% identical repeat sequences with a minimum repeat size of 30 bp with a hamming distance equal to 3 (i.e., the gap size between repeats had a maximum length of 3 bp). All overlapping repeats were detached from the final consequences, whose repeating sequence regions were between 1 and 5 bp and repeated no less than three times were considered as SSRs. Besides, the length of repeats ≥ 20 bp were considered as large repeat sequences. The Cp SSRs were detected by MISA^65^ with search parameters of 10 repeat units for mononucleotide SSRs, five repeat units for dinucleotide SSRs, four repeat units for trinucleotide SSRs, and three repeat units for tetra-, penta-, and hexanucleotide SSRs, respectively.

### Prediction of RNA editing sites and codon usage analysis

The Prep-Cp was developed to computationally identify RNA editing sites.^66^ For the analysis, the threshold value was set to 0.8 to ensure the accuracy of the prediction. To claim the consequences of codon usage bias, we applied the program CodonW1.4.2^67^ to analyze the synonymous codon preference of protein-coding genes shorter than 300 bp in length. Each CDS contains initiation codon and termination codon. RSCU is a qualified frequency that synonymous with every codon was specific to encoding an amino acid.

### Comparison and analysis among genomes

The complete genomes of six species were compared by mVISTA,^68^ including *A. thaliana, P. grandifloras*, and four species in Plantaginaceae *(P. asiatica, P. depressa, P. lagopus*, and *P. ovata)*. IRscope^69^ was also employed to compare the borders of four main regions in the chloroplast genomes of the six species.

### Phylogenetic position analysis

To detect divergence among the genus *Plantago* Cp genomes and its associated species, 17 species (including *P. asiatica*) were downloaded from the NCBI database to study the coding region evolution. Entire chloroplast genomes were used to examine these species’ average pairwise sequence divergence. Phylogenetic analysis was done after all related sequences were first aligned using MAFFT under default parameters.^70^ Alignments were trimmed using trimAI (v.1.2).^71^ The BIC value was used to predict the best fitting model GTRGAMMAX to perform the ML analysis by ModelTest-NG (v.0.1.4).^72^ The final maximum likelihood tree was constructed by RAxML (v. 7.7.8)^73^ with 1000 rapid bootstrap, which was rooted by an outgroup of *A. thaliana*. In addition to this, BI was employed in MrBayes (v.3.1.2). The Markov Chain Monte Carlo (MCMC) method was run using four incrementally heated chains across 1,000,000 generations, starting from random trees and sampling 1 out of every 100 generations. Pi and sequence polymorphism of the specific 18 species were analyzed using DnaSP (v 5.10.1).^74^

## Conclusion

In this study, we generated the chloroplast genome of *P. asiatica* with a classical tetrameric structure of 164, 992 bp in length, including 8 rRNA, 38 tRNA, and 95 protein-coding genes. Among all genes, 28,949 codons had G or C terminals. There were 47 editing sites in 16 genes, all of which were C to T conversions. Serine (S) transformation into leucine (L) can increase protein hydrophobicity. Significantly, we detected 72 SSRs in *P. asiatica* that can be utilized in DNA barcoding to distinguish similar species. The *P. asiatica* chloroplast genome shared similar overall organization and gene contents with most chloroplast genomes, including the closest species. The phylogeny analysis showed that *P. asiatica* formed a sister to *P. depressa*. Besides, Plantaginaceae was closer to Gesneriaceae and Scrophulariaceae in Lamiales than Plumbaginaceae in Caryophyllales. Our genome comparison analysis revealed highly conserved gene sequences and several differences among Plantaginaceae species, which can assist us in further studying these evolutionary relationships. Species in the family Plantaginaceae are usually difficult to distinguish due to similarities in phenotype, while different metabolites in different plants have various effects. So, these conserved gene sequences in the chloroplast genome can be used to identify species of Plantaginaceae. Our findings not only provide new insights into chloroplast genomic and phylogenetic relationships of *P. asiatica* but also will assist with maximizing further development and application of this plant.

## Supplementary Material

Supplemental Material

## Data Availability

The data that support the findings of this study are openly available in GenBank of NCBI (https://www.ncbi.nlm.nih.gov) under the access number OL694048. The associated BioProject, SRA and Bio-Sample numbers are PRJNA797537, SRR17629686, and S SAMN25008951 respectively.
