# Supplementary material for: Comprehensive analysis of complete chloroplast genome sequence of Plantago asiatica L. (Plantaginaceae)
Source: Plant Signal Behav. 2023 Jan 2;18(1):2163345. doi: 10.1080/15592324.2022.2163345 (PMC9809945; doi:10.1080/15592324.2022.2163345)
Supplement: Supplemental Material [file KPSB_A_2163345_SM8122.zip › Supplement materials.pdf]

Supplement materials

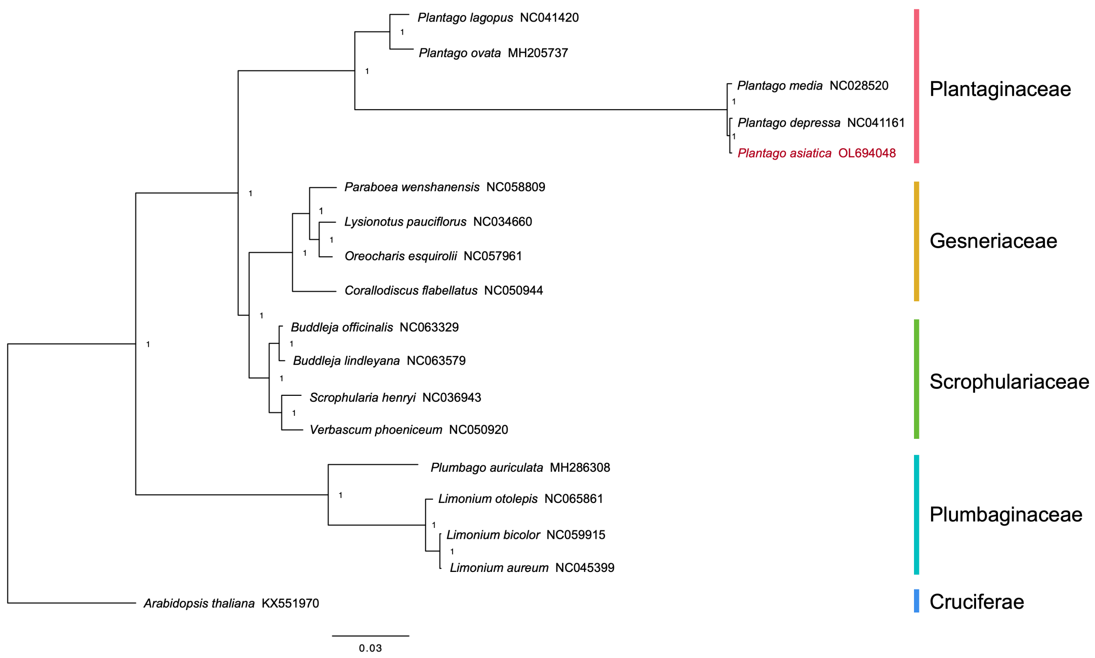

Figure S1. Bayesian inference tree based on chloroplast sequences in the study.

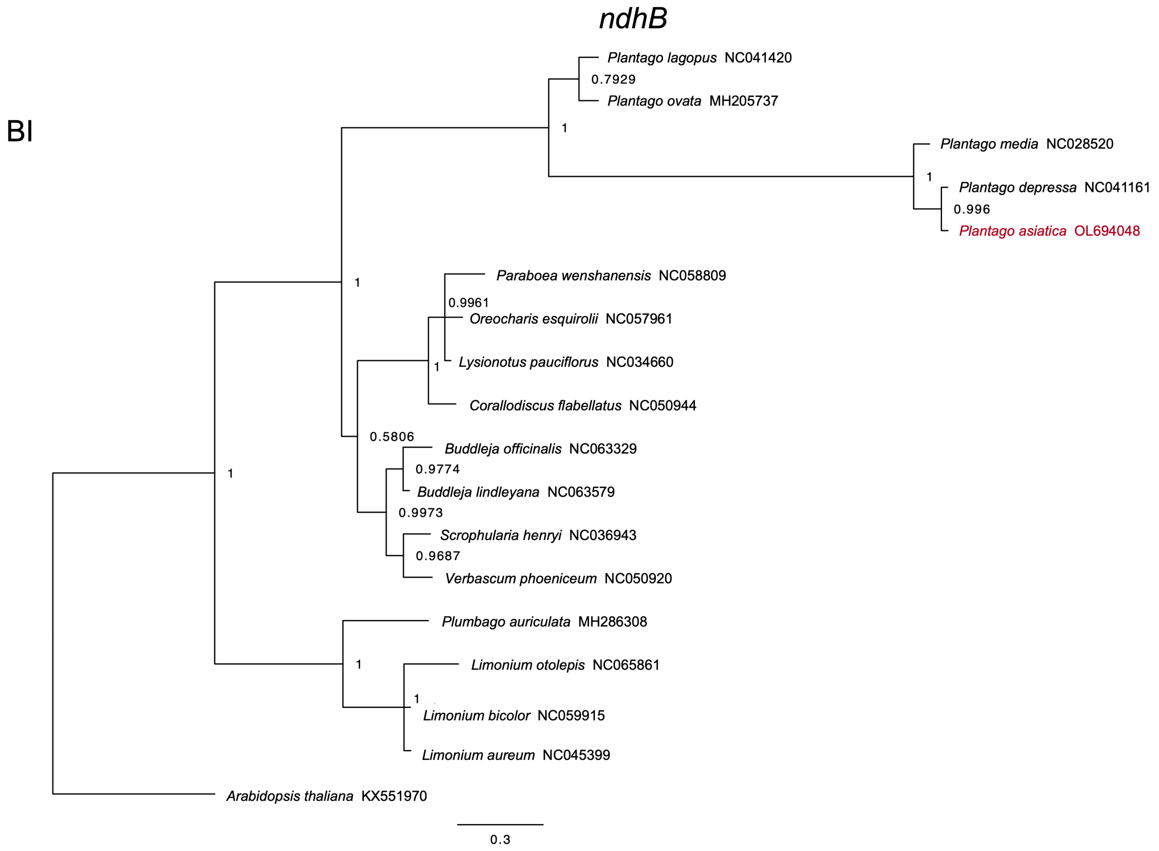

Figure S2. A. Bayesian inference trees based on *ndhB* gene sequences.

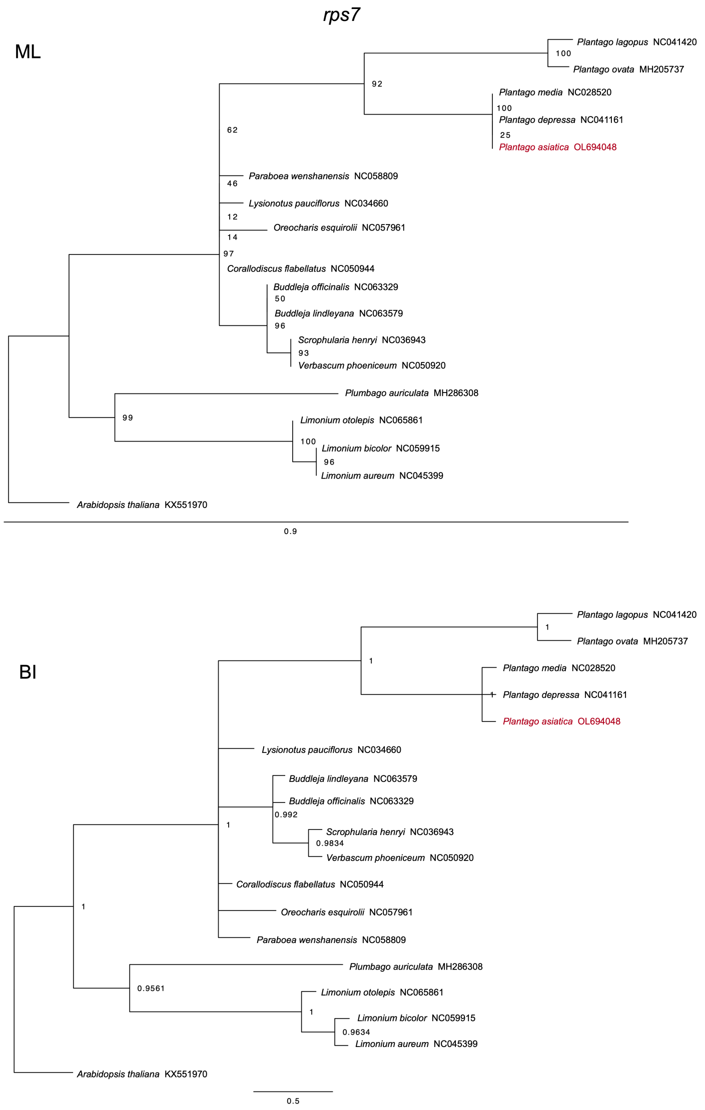

Figure S3. A. Maximum likelihood and Bayesian inference trees based on *rps7* gene sequences.

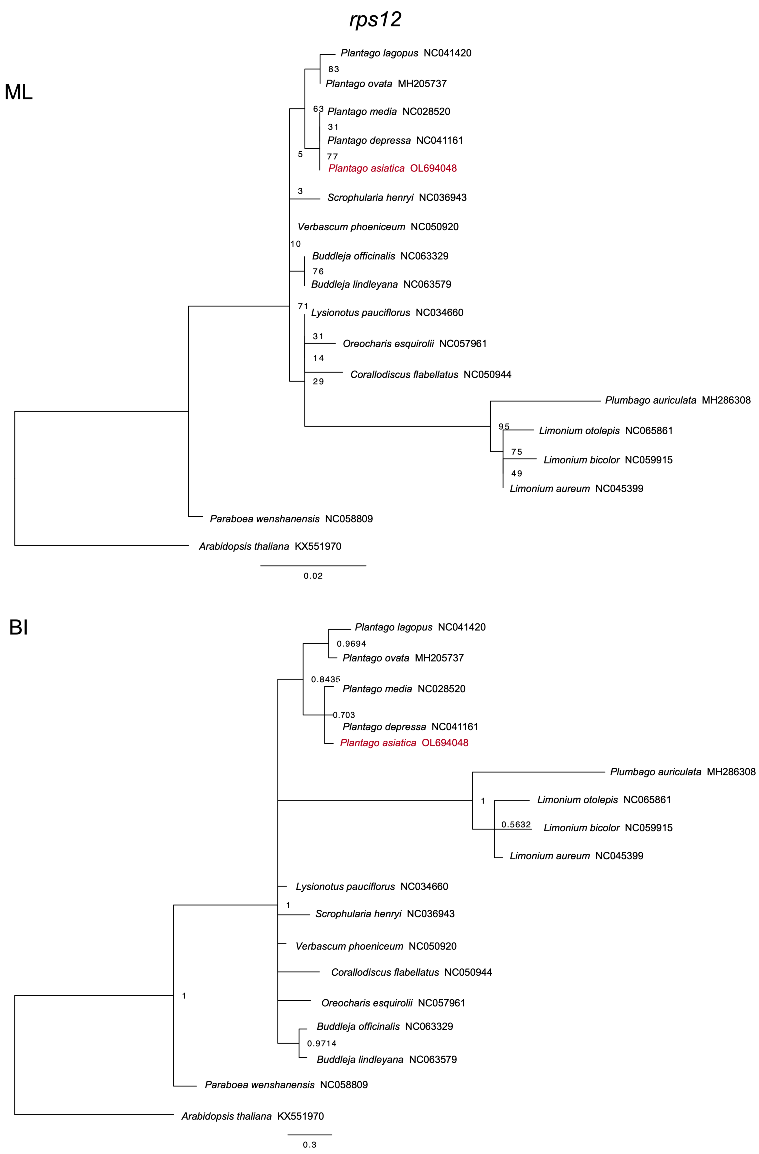

Figure S4. A. Maximum likelihood and Bayesian inference trees based on *rps12* gene sequences.

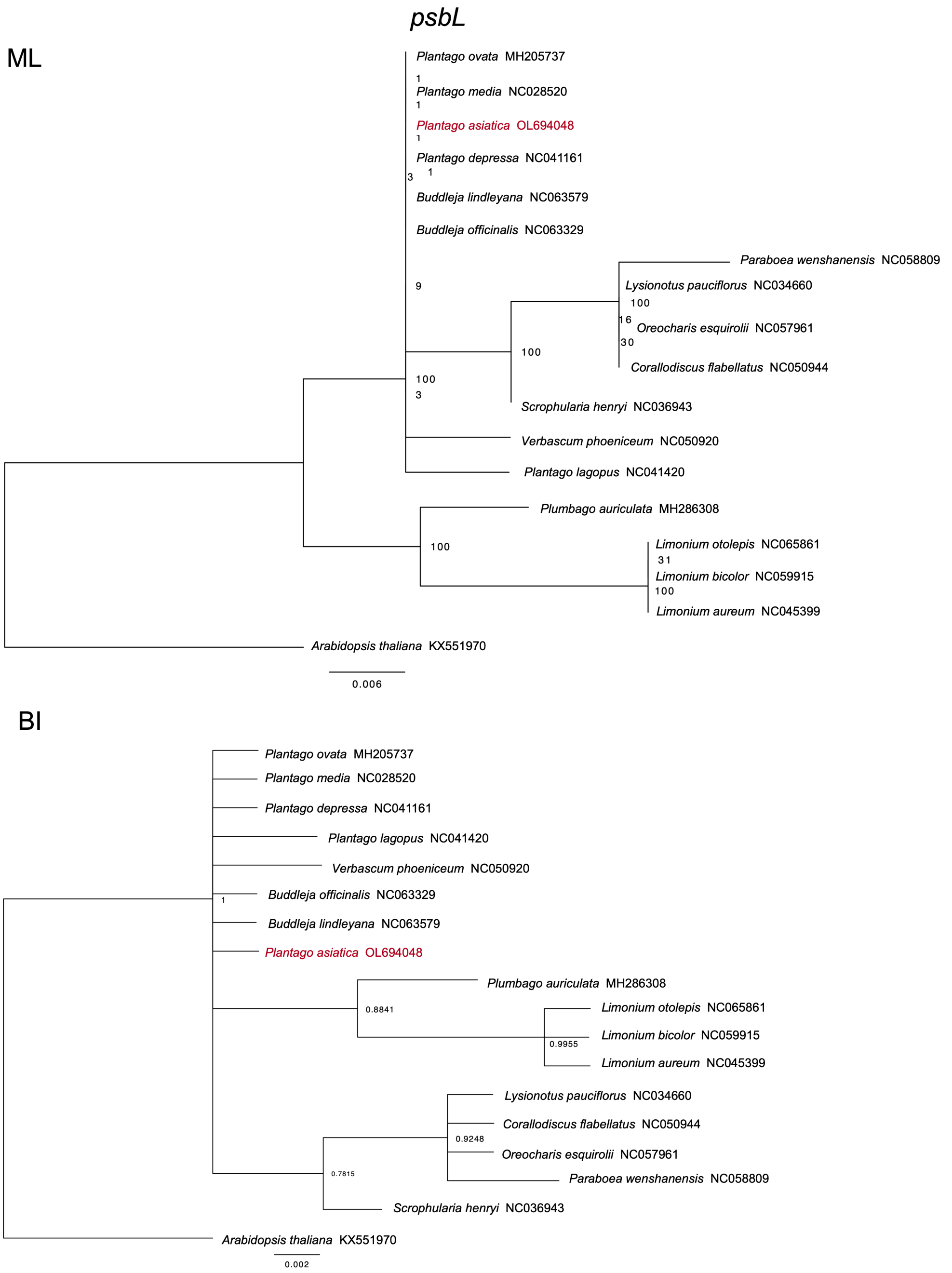

Figure S5. Maximum likelihood and Bayesian inference trees based on *psbL* gene sequences.
