# Supplementary material for: Comprehensive analysis of complete chloroplast genome sequence of Plantago asiatica L. (Plantaginaceae)
Source: Plant Signal Behav. 2023 Jan 2;18(1):2163345. doi: 10.1080/15592324.2022.2163345 (PMC9809945; doi:10.1080/15592324.2022.2163345)
Supplement: Supplemental Material [file KPSB_A_2163345_SM8122.zip › Supplementary Tables.pdf]

Supplementary Table S1

**Genes in chloroplast genome of *Plantago asiatica***			
**Gene**	**Frequency of occurrence**	**Location**	*** Gene containing introns**
accD		LSC	
atpA	2	LSC	
atpB		LSC	
atpE		LSC	
atpF		LSC	*
atpH		LSC	
atpl		LSC	
ccsA	2	SSC;IRA	
cemA		LSC	
clpP1		LSC	*
infA		LSC	
matK		LSC	
ndhA	1;1	IRA;IRB	*
ndhB	1;1	IRA;IRB	*
ndhC		LSC	
ndhD	1;1	IRA;IRB	
ndhE	1;1	IRA;IRB	
ndhF	2	SSC	
ndhG	1;1	IRA;IRB	
ndhH	1;1	IRA;IRB	
ndhJ		LSC	
ndhK		LSC	
ndhl	1;1	IRA;IRB	
ycf3		LSC	*
ycf4		LSC	
psbN	2	LSC	
petA		LSC	
petB	2	LSC	*
petD		LSC	*
petG		LSC	
petL		LSC	
petN		LSC	
psaA		LSC	
psaB		LSC	
psaC	1;1	IRA;IRB	
psaJ		LSC	
psal		LSC	
psbA		LSC	

psbB		LSC	
psbC		LSC	
psbD	2	LSC	
psbE		LSC	
psbF		LSC	
psbH	2	LSC	
psbJ		LSC	
psbK		LSC	
psbL		LSC	
psbI		LSC	
psbM		LSC	
psbT		LSC	
psbZ		LSC	
rbcL		LSC	
rpl14	2	LSC	
rpl16		LSC	*
rpl2	1;1	IRA;IRB	*
rpl20	2	LSC	
rpl22		LSC	
rpl23	1;1	IRA;IRB	
rpl32		SSC	
rpl33		LSC	
rpl36		LSC	
rpoA		LSC	
rpoB		LSC	
rpoC1		LSC	*
rpoC2		LSC	
rps11		LSC	
rps12	3	IRA;IRB;LSC	*
rps14		LSC	
rps15	1;1	IRA;IRB	
rps16		LSC	*
rps18		LSC	
rps19	1;1	LSC	
rps2		LSC	
rps3		LSC	
rps4		LSC	
rps7	1;1	IRA;IRB	
rps8		LSC	
rrn16	1;1	IRA;IRB	
rrn23	2;2	IRA;IRB	

rrn4.5	1;1	IRA;IRB	
rrn5	1;1	IRA;IRB	
trnA-UGC	1;1	IRA;IRB	*
trnC-GCA		LSC	
trnD-GUC		LSC	
trnE-UUC		LSC	
trnF-GAA		LSC	
trnG-GCC		LSC	
trnG-UCC		LSC	*
trnH-GUG	2	LSC	
trnK-UUU		LSC	*
trnL-CAA	1;1;1	IRA;IRB;LSC	
trnl-GAU	2;2	IRA;IRB	*
trnL-UAA		LSC	*
trnL-UAG		SSC	
trnM-CAU / trnl-CAU	1;1;1	IRA;IRB;LSC	
trnN-GUU	1;1	IRA;IRB	
trnP-UGG		LSC	
trnQ-UUG		LSC	
trnR-ACG	1;1	IRA;IRB	
trnR-UCU		LSC	
trnS-GCU		LSC	
trnS-GGA		LSC	
trnS-UGA		LSC	
trnT-GGU		LSC	
trnT-UGU		LSC	
trnV-GAC	1;1	IRA;IRB	
trnV-UAC		LSC	*
trnW-CCA		LSC	
trnY-GUA		LSC	
ycf1	1;1	IRA;IRB	
ycf2	2;2	IRA;IRB	
**Gene containing intron*			
LSC, large single copy; SSC, small single copy; IR, inverted repeat			

Supplementary Table S2

**The NR annotation of genes in chloroplast ggenome of *P. asiatica***					
Gene ID	ID	Description	Identity	Evalue	Score
rps12	YP_009305573.1	ribosomal protein S12 (plastid) [Veronica persica]	96.7	3.70E-59	236.9
rps12	YP_009305573.1	ribosomal protein S12 (plastid) [Veronica persica]	96.7	3.70E-59	236.9
psbA	YP_009365640.1	photosystem II protein D1 precursor (plastid) [Digitalis lanata]	99.7	7.20E-204	719.2
matK	YP_009183060.1	maturase K (plastid) [Plantago media]	99	4.80E-294	1019
psbK	YP_009183062.1	photosystem II protein K (plastid) [Plantago media]	100	3.00E-25	123.2
psbI	RDY14692.1	Photosystem II reaction center protein K, partial [Mucuna pruriens]	100	3.40E-11	75.9
atpA	YP_009183064.1	ATP synthase CF1 alpha subunit (plastid) [Plantago media]	100	1.60E-276	961.1
atpF	YP_009183065.1	ATP synthase CF0 subunit I (plastid) [Plantago media]	98.4	1.80E-92	348.2
atpH	OAY68049.1	ATP synthase subunit c, chloroplastic [Ananas comosus]	100	3.70E-34	153.3
atpI	YP_009183067.1	ATP synthase CF0 subunit IV (plastid) [Plantago media]	100	2.00E-136	494.6
rps2	YP_009183068.1	ribosomal protein S2 (plastid) [Plantago media]	99.6	1.20E-133	485.3
rpoC2	YP_009183069.1	RNA polymerase beta' subunit (plastid) [Plantago media]	99.1	0.00E+00	2735
rpoC1	YP_009183070.1	RNA polymerase beta (plastid) [Plantago media]	100	0.00E+00	1398
rpoB	YP_009183071.1	RNA polymerase beta subunit (plastid) [Plantago media]	99.7	0.00E+00	2133
petN	WP_069187528.1	cytochrome b6-f complex subunit PetN [Staphylococcus aureus]	100	1.50E-06	60.1
psbM	ADD63027.1	photosystem II protein M (chloroplast) [Potamophila parviflora]	100	1.90E-08	66.6
psbD	YP_009182984.1	photosystem II protein D2 (plastid) [Plantago maritima]	100	1.30E-208	734.9

psbC	YP_009183075.1	photosystem II CP43 chlorophyll apoprotein (plastid) [Plantago media]	99.8	3.60E-283	983
psbZ	YP_778487.1	PSII Z protein [Jasminum nudiflorum]	100	2.60E-24	120.2
rps14	YP_009183077.1	ribosomal protein S14 (plastid) [Plantago media]	100	1.30E-49	204.9
psaB	YP_009183078.1	photosystem I P700 apoprotein A2 (plastid) [Plantago media]	100	0.00E+00	1545
psaA	YP_009183079.1	photosystem I P700 apoprotein A1 (plastid) [Plantago media]	100	0.00E+00	1559
ycf3	YP_009182990.1	hypothetical chloroplast RF34 (plastid) [Plantago maritima]	100	2.70E-92	347.4
rps4	YP_009183081.1	ribosomal protein S4 (plastid) [Plantago media]	100	8.60E-109	402.5
ndhJ	YP_009183082.1	NADH-plastoquinone oxidoreductase subunit J (plastid) [Plantago media]	100	4.50E-89	336.7
ndhK	YP_009183083.1	NADH-plastoquinone oxidoreductase subunit K (plastid) [Plantago media]	100	3.00E-126	460.7
ndhC	YP_004376426.1	NADH dehydrogenase ND3 subunit [Olea europaea subsp. europaea]	99.2	8.80E-61	242.3
atpE	YP_009183085.1	ATP synthase CF1 epsilon subunit (plastid) [Plantago media]	100	7.70E-66	259.2
atpB	YP_009183086.1	ATP synthase CF1 beta subunit (plastid) [Plantago media]	99.6	5.30E-277	962.6
rbcL	YP_009365668.1	ribulose-1,5-bisphosphate caroxylase/oxygenase (plastid) [Digitalis lanata]	99.4	9.50E-284	984.9
accD	YP_009183088.1	acetyl-CoA carboxylase carboxyltransferase beta subunit (plastid) [Plantago media]	95	1.10E-244	855.1
psaI	YP_007507121.1	photosystem I subunit VIII (chloroplast) [Salvia miltiorrhiza]	97.2	1.30E-10	73.9
ycf4	YP_009183090.1	photosystem I assembly protein ycf4 (plastid) [Plantago media]	96.2	5.80E-96	359.8
cemA	YP_009183091.1	chloroplast envelope membrane protein (plastid) [Plantago media]	99.6	2.70E-127	464.2
petA	YP_009183092.1	cytochrome f (plastid) [Plantago media]	99.7	4.30E-179	636.7

psbJ	WP_110286114.1	photosystem II reaction center protein J [Halomonas heilongjiangensis]	100	2.30E-13	83.2
psbL	RCU61563.1	hypothetical protein SETIT_J013200v2 [Setaria italica]	100	1.10E-12	80.9
psbF	NP_054516.1	photosystem II protein VI [Nicotiana tabacum]	100	5.10E-13	82
psbE	YP_003934324.1	photosystem II cytochrome b559 alpha subunit [Monsonia speciosa]	100	1.00E-39	171.8
petL	YP_784083.1	cytochrome b6/f complex subunit VI [Pelargonium x hortorum]	100	1.60E-06	60.1
petG	YP_009183008.1	cytochrome b6/f complex subunit V (plastid) [Plantago maritima]	100	5.90E-11	75.1
psaJ	YP_009256054.1	photosystem I subunit IX (chloroplast) [Scopolia parviflora]	100	5.50E-16	92
rpl33	YP_009183100.1	ribosomal protein L33 (plastid) [Plantago media]	100	4.00E-31	142.9
rps18	YP_009183101.1	ribosomal protein S18 (plastid) [Plantago media]	99.2	6.40E-62	246.1
rpl20	YP_009183102.1	ribosomal protein L20 (plastid) [Plantago media]	100	4.80E-57	229.9
clpP1	YP_009183104.1	clp protease proteolytic subunit (plastid) [Plantago media]	99.5	6.70E-106	392.9
psbB	YP_009183105.1	photosystem II CP47 chlorophyll apoprotein (plastid) [Plantago media]	100	1.50E-306	1061
psbT	YP_009494316.1	PsbT (chloroplast) [Dendrosenecio kilimanjari]	100	1.10E-08	67.4
psbN	YP_009183107.1	photosystem II protein N (plastid) [Plantago media]	100	2.70E-15	89.7
psbH	YP_009183108.1	photosystem II phosphoprotein (plastid) [Plantago media]	100	5.30E-32	146
rpoA	YP_009183111.1	RNA polymerase alpha subunit (plastid) [Plantago media]	99.4	9.60E-190	672.2
rps11	YP_009183112.1	ribosomal protein S11 (plastid) [Plantago media]	99.3	2.90E-71	277.3
rpl36	YP_567111.1	ribosomal protein L36 (chloroplast) [Vitis vinifera]	100	1.80E-12	80.1
infA	YP_009183114.1	translational initiation factor 1 (plastid) [Plantago media]	100	3.20E-35	156.8
rps8	YP_009183115.1	ribosomal protein S8 (plastid) [Plantago media]	100	4.10E-67	263.5

rpl14	YP_004563816.1	ribosomal protein L14 [Olea europaea subsp. cuspidata]	99.2	5.80E-60	239.6
rps3	YP_009183118.1	ribosomal protein S3 (plastid) [Plantago media]	100	6.80E-123	449.5
rpl22	YP_009183119.1	ribosomal protein L22 (plastid) [Plantago media]	100	5.30E-91	343.2
rps19	YP_009183120.1	ribosomal protein S19 (plastid) [Plantago media]	96.7	4.40E-44	186.4
rpl2	YP_009183121.1	ribosomal protein L2 (plastid) [Plantago media]	100	8.80E-157	562.4
rpl23	YP_009183122.1	ribosomal protein L23 (plastid) [Plantago media]	100	1.60E-46	194.5
rps12	YP_009305573.1	ribosomal protein S12 (plastid) [Veronica persica]	96.7	3.70E-59	236.9
ycf2	YP_009183123.1	hypothetical chloroplast RF21 (plastid) [Plantago media]	99.9	0.00E+00	3785
rps7	YP_009183124.1	ribosomal protein S7 (plastid) [Plantago media]	100	3.50E-78	300.4
ndhB	YP_009183125.1	NADH-plastoquinone oxidoreductase subunit 2 (plastid) [Plantago media]	99.4	2.30E-277	963.8
ycf1	YP_009183126.1	hypothetical chloroplast RF19 (plastid) [Plantago media]	98.7	0.00E+00	3902
rps15	YP_009183127.1	ribosomal protein S15 (plastid) [Plantago media]	100	1.30E-40	174.9
ndhH	YP_009183128.1	NADH-plastoquinone oxidoreductase subunit 7 (plastid) [Plantago media]	100	3.50E-231	810.1
ndhA	YP_009183129.1	NADH dehydrogenase subunit 1 (plastid) [Plantago media]	100	1.20E-198	701.8
ndhI	YP_009183130.1	NADH-plastoquinone oxidoreductase subunit I (plastid) [Plantago media]	100	1.40E-93	351.7
ndhG	YP_009183131.1	NADH-plastoquinone oxidoreductase subunit 6 (plastid) [Plantago media]	100	4.50E-93	350.1
ndhE	YP_009183132.1	NADH-plastoquinone oxidoreductase subunit 4L (plastid) [Plantago media]	100	6.80E-46	192.6
psaC	KYP33292.1	Photosystem I iron-sulfur center [Cajanus cajan]	100	2.10E-42	180.6

ndhD	YP_009183134.1	NADH-plastoquinone oxidoreductase subunit 4 (plastid) [Plantago media]	100	2.40E-293	1017
ndhF	YP_009183135.1	NADH-plastoquinone oxidoreductase subunit 5 (plastid) [Plantago media]	97.7	0.00E+00	1459
rpl32	YP_009183042.1	ribosomal protein L32 (plastid) [Plantago maritima]	98.1	8.20E-22	111.7
ccsA	YP_009183137.1	cytochrome c heme attachment protein (plastid) [Plantago media]	96.2	1.60E-173	618.2
ndhD	YP_009183134.1	NADH-plastoquinone oxidoreductase subunit 4 (plastid) [Plantago media]	100	2.40E-293	1017
psaC	KYP33292.1	Photosystem I iron-sulfur center [Cajanus cajan]	100	2.10E-42	180.6
ndhE	YP_009183132.1	NADH-plastoquinone oxidoreductase subunit 4L (plastid) [Plantago media]	100	6.80E-46	192.6
ndhG	YP_009183131.1	NADH-plastoquinone oxidoreductase subunit 6 (plastid) [Plantago media]	100	4.50E-93	350.1
ndhI	YP_009183130.1	NADH-plastoquinone oxidoreductase subunit I (plastid) [Plantago media]	100	1.40E-93	351.7
ndhA	YP_009183129.1	NADH dehydrogenase subunit 1 (plastid) [Plantago media]	100	1.20E-198	701.8
ndhH	YP_009183128.1	NADH-plastoquinone oxidoreductase subunit 7 (plastid) [Plantago media]	100	3.50E-231	810.1
rps15	YP_009183127.1	ribosomal protein S15 (plastid) [Plantago media]	100	1.30E-40	174.9
ycf1	YP_009183126.1	hypothetical chloroplast RF19 (plastid) [Plantago media]	98.7	0.00E+00	3902
ndhB	YP_009183125.1	NADH-plastoquinone oxidoreductase subunit 2 (plastid) [Plantago media]	99.4	2.30E-277	963.8
rps7	YP_009183124.1	ribosomal protein S7 (plastid) [Plantago media]	100	3.50E-78	300.4
ycf2	YP_009183123.1	hypothetical chloroplast RF21 (plastid) [Plantago media]	99.9	0.00E+00	3785
rps12	YP_009305573.1	ribosomal protein S12 (plastid) [Veronica persica]	96.7	3.70E-59	236.9

rpl23	YP_009183122.1	ribosomal protein L23 (plastid) [Plantago media]	100	1.60E-46	194.5
rpl2	YP_009183121.1	ribosomal protein L2 (plastid) [Plantago media]	100	8.80E-157	562.4

Supplementary Table S3

The GO classification of genes based on three categories			
**Gene_ID**	**GO_ID**	**GO_terms**	**GO_Aspect**
accD	GO:0003989	acetyl-CoA carboxylase activity	molecular_function
accD	GO:0006633	fatty acid biosynthetic process	biological_process
accD	GO:0009317	acetyl-CoA carboxylase complex	cellular_component
atpA	GO:0005524	ATP binding	molecular_function
atpA	GO:0015986	ATP synthesis coupled proton transport	biological_process
atpA	GO:0032559	adenyl ribonucleotide binding	molecular_function
atpA	GO:0045261	proton-transporting ATP synthase complex, catalytic core F(1)	cellular_component
atpA	GO:0046034	ATP metabolic process	biological_process
atpA	GO:0046933	proton-transporting ATP synthase activity, rotational mechanism	molecular_function
atpA	GO:1902600	proton transmembrane transport	biological_process
atpB	GO:0005524	ATP binding	molecular_function
atpB	GO:0015986	ATP synthesis coupled proton transport	biological_process
atpB	GO:0045261	proton-transporting ATP synthase complex, catalytic core F(1)	cellular_component
atpB	GO:0046034	ATP metabolic process	biological_process
atpB	GO:0046933	proton-transporting ATP synthase activity, rotational mechanism	molecular_function
atpB	GO:1902600	proton transmembrane transport	biological_process
atpE	GO:0015986	ATP synthesis coupled proton transport	biological_process
atpE	GO:0045261	proton-transporting ATP synthase complex, catalytic core F(1)	cellular_component
atpE	GO:0046933	proton-transporting ATP synthase activity, rotational mechanism	molecular_function
atpH	GO:0015078	proton transmembrane transporter activity	molecular_function
atpH	GO:0015986	ATP synthesis coupled proton transport	biological_process
atpH	GO:0015991	ATP hydrolysis coupled proton transport	biological_process
atpH	GO:0033177	proton-transporting two-sector ATPase complex, proton-transporting domain	cellular_component
atpH	GO:0045263	proton-transporting ATP synthase complex, coupling factor F(o)	cellular_component
atpI	GO:0015078	proton transmembrane transporter activity	molecular_function
atpI	GO:0015986	ATP synthesis coupled proton transport	biological_process
atpI	GO:0045263	proton-transporting ATP synthase complex, coupling factor F(o)	cellular_component
ccsA	GO:0017004	cytochrome complex assembly	biological_process
ccsA	GO:0020037	heme binding	molecular_function

cemA	GO:0016021	integral component of membrane	cellular_component
clpP1	GO:0004252	serine-type endopeptidase activity	molecular_function
clpP1	GO:0006508	proteolysis	biological_process
infA	GO:0003723	RNA binding	molecular_function
infA	GO:0003743	translation initiation factor activity	molecular_function
infA	GO:0006413	translational initiation	biological_process
matK	GO:0006397	mRNA processing	biological_process
matK	GO:0009507	chloroplast	cellular_component
ndhA	GO:0016020	membrane	cellular_component
ndhA	GO:0055114	oxidation-reduction process	biological_process
ndhB	GO:0008137	NADH dehydrogenase (ubiquinone) activity	molecular_function
ndhB	GO:0042773	ATP synthesis coupled electron transport	biological_process
ndhB	GO:0055114	oxidation-reduction process	biological_process
ndhC	GO:0008137	NADH dehydrogenase (ubiquinone) activity	molecular_function
ndhC	GO:0016651	oxidoreductase activity, acting on NAD(P)H	molecular_function
ndhC	GO:0055114	oxidation-reduction process	biological_process
ndhD	GO:0008137	NADH dehydrogenase (ubiquinone) activity	molecular_function
ndhD	GO:0016655	oxidoreductase activity, acting on NAD(P)H, quinone or similar compound as acceptor	molecular_function
ndhD	GO:0042773	ATP synthesis coupled electron transport	biological_process
ndhD	GO:0055114	oxidation-reduction process	biological_process
ndhE	GO:0016651	oxidoreductase activity, acting on NAD(P)H	molecular_function
ndhE	GO:0042773	ATP synthesis coupled electron transport	biological_process
ndhE	GO:0055114	oxidation-reduction process	biological_process
ndhG	GO:0008137	NADH dehydrogenase (ubiquinone) activity	molecular_function
ndhG	GO:0055114	oxidation-reduction process	biological_process
ndhH	GO:0016651	oxidoreductase activity, acting on NAD(P)H	molecular_function
ndhH	GO:0048038	quinone binding	molecular_function
ndhH	GO:0051287	NAD binding	molecular_function
ndhH	GO:0055114	oxidation-reduction process	biological_process
ndhI	GO:0008137	NADH dehydrogenase (ubiquinone) activity	molecular_function
ndhI	GO:0016020	membrane	cellular_component
ndhI	GO:0016651	oxidoreductase activity, acting on NAD(P)H	molecular_function
ndhI	GO:0051539	4 iron, 4 sulfur cluster binding	molecular_function
ndhI	GO:0055114	oxidation-reduction process	biological_process
ndhJ	GO:0008137	NADH dehydrogenase (ubiquinone) activity	molecular_function
ndhJ	GO:0016651	oxidoreductase activity, acting on NAD(P)H	molecular_function
ndhJ	GO:0055114	oxidation-reduction process	biological_process
ndhK	GO:0008137	NADH dehydrogenase (ubiquinone) activity	molecular_function
ndhK	GO:0048038	quinone binding	molecular_function
ndhK	GO:0051536	iron-sulfur cluster binding	molecular_function

ndhK	GO:0051539	4 iron, 4 sulfur cluster binding	molecular_function
ndhK	GO:0055114	oxidation-reduction process	biological_process
ycf3	GO:0005515	protein binding	molecular_function
ycf4	GO:0009522	photosystem I	cellular_component
ycf4	GO:0009579	thylakoid	cellular_component
ycf4	GO:0015979	photosynthesis	biological_process
ycf4	GO:0016021	integral component of membrane	cellular_component
psbN	GO:0009523	photosystem II	cellular_component
psbN	GO:0009539	photosystem II reaction center	cellular_component
psbN	GO:0015979	photosynthesis	biological_process
psbN	GO:0016020	membrane	cellular_component
petA	GO:0005506	iron ion binding	molecular_function
petA	GO:0009055	electron transfer activity	molecular_function
petA	GO:0015979	photosynthesis	biological_process
petA	GO:0020037	heme binding	molecular_function
petA	GO:0031361	integral component of thylakoid membrane	cellular_component
petG	GO:0009512	cytochrome b6f complex	cellular_component
petL	GO:0009055	electron transfer activity	molecular_function
petL	GO:0009512	cytochrome b6f complex	cellular_component
petN	GO:0009512	cytochrome b6f complex	cellular_component
petN	GO:0017004	cytochrome complex assembly	biological_process
petN	GO:0045158	electron transporter, transferring electrons within cytochrome b6/f complex of photosystem II activity	molecular_function
psaA	GO:0009522	photosystem I	cellular_component
psaA	GO:0009579	thylakoid	cellular_component
psaA	GO:0015979	photosynthesis	biological_process
psaA	GO:0016021	integral component of membrane	cellular_component
psaA	GO:0046872	metal ion binding	molecular_function
psaB	GO:0009522	photosystem I	cellular_component
psaB	GO:0009579	thylakoid	cellular_component
psaB	GO:0015979	photosynthesis	biological_process
psaB	GO:0016021	integral component of membrane	cellular_component
psaC	GO:0009055	electron transfer activity	molecular_function
psaC	GO:0009522	photosystem I	cellular_component
psaC	GO:0009773	photosynthetic electron transport in photosystem I	biological_process
psaC	GO:0015979	photosynthesis	biological_process
psaC	GO:0042651	thylakoid membrane	cellular_component
psaC	GO:0051539	4 iron, 4 sulfur cluster binding	molecular_function
psaI	GO:0009522	photosystem I	cellular_component

psaI	GO:0015979	photosynthesis	biological_process
psaJ	GO:0009522	photosystem I	cellular_component
psaJ	GO:0015979	photosynthesis	biological_process
psbA	GO:0009055	electron transfer activity	molecular_function
psbA	GO:0009772	photosynthetic electron transport in photosystem II	biological_process
psbA	GO:0019684	photosynthesis, light reaction	biological_process
psbA	GO:0045156	electron transporter, transferring electrons within the cyclic electron transport pathway of photosynthesis activity	molecular_function
psbB	GO:0009521	photosystem	cellular_component
psbB	GO:0009523	photosystem II	cellular_component
psbB	GO:0009767	photosynthetic electron transport chain	biological_process
psbB	GO:0009772	photosynthetic electron transport in photosystem II	biological_process
psbB	GO:0015979	photosynthesis	biological_process
psbB	GO:0016020	membrane	cellular_component
psbB	GO:0016168	chlorophyll binding	molecular_function
psbB	GO:0019684	photosynthesis, light reaction	biological_process
psbB	GO:0045156	electron transporter, transferring electrons within the cyclic electron transport pathway of photosynthesis activity	molecular_function
psbC	GO:0009521	photosystem	cellular_component
psbC	GO:0009523	photosystem II	cellular_component
psbC	GO:0009767	photosynthetic electron transport chain	biological_process
psbC	GO:0009772	photosynthetic electron transport in photosystem II	biological_process
psbC	GO:0015979	photosynthesis	biological_process
psbC	GO:0016020	membrane	cellular_component
psbC	GO:0016168	chlorophyll binding	molecular_function
psbC	GO:0019684	photosynthesis, light reaction	biological_process
psbC	GO:0045156	electron transporter, transferring electrons within the cyclic electron transport pathway of photosynthesis activity	molecular_function
psbD	GO:0009523	photosystem II	cellular_component
psbD	GO:0009772	photosynthetic electron transport in photosystem II	biological_process
psbD	GO:0015979	photosynthesis	biological_process
psbD	GO:0016020	membrane	cellular_component
psbD	GO:0019684	photosynthesis, light reaction	biological_process

psbD	GO:0045156	electron transporter, transferring electrons within the cyclic electron transport pathway of photosynthesis activity	molecular_function
psbE	GO:0009523	photosystem II	cellular_component
psbE	GO:0009539	photosystem II reaction center	cellular_component
psbE	GO:0009767	photosynthetic electron transport chain	biological_process
psbE	GO:0015979	photosynthesis	biological_process
psbE	GO:0016021	integral component of membrane	cellular_component
psbE	GO:0019684	photosynthesis, light reaction	biological_process
psbE	GO:0020037	heme binding	molecular_function
psbE	GO:0046872	metal ion binding	molecular_function
psbF	GO:0009523	photosystem II	cellular_component
psbF	GO:0009539	photosystem II reaction center	cellular_component
psbF	GO:0009767	photosynthetic electron transport chain	biological_process
psbF	GO:0015979	photosynthesis	biological_process
psbF	GO:0016021	integral component of membrane	cellular_component
psbF	GO:0019684	photosynthesis, light reaction	biological_process
psbF	GO:0020037	heme binding	molecular_function
psbH	GO:0009523	photosystem II	cellular_component
psbH	GO:0015979	photosynthesis	biological_process
psbH	GO:0016020	membrane	cellular_component
psbH	GO:0042301	phosphate ion binding	molecular_function
psbH	GO:0050821	protein stabilization	biological_process
psbI	GO:0009523	photosystem II	cellular_component
psbI	GO:0009539	photosystem II reaction center	cellular_component
psbI	GO:0015979	photosynthesis	biological_process
psbI	GO:0016020	membrane	cellular_component
psbJ	GO:0009523	photosystem II	cellular_component
psbJ	GO:0009539	photosystem II reaction center	cellular_component
psbJ	GO:0015979	photosynthesis	biological_process
psbJ	GO:0016020	membrane	cellular_component
psbK	GO:0009523	photosystem II	cellular_component
psbK	GO:0009539	photosystem II reaction center	cellular_component
psbK	GO:0015979	photosynthesis	biological_process
psbL	GO:0009523	photosystem II	cellular_component
psbL	GO:0009539	photosystem II reaction center	cellular_component
psbL	GO:0015979	photosynthesis	biological_process
psbL	GO:0016020	membrane	cellular_component
psbM	GO:0009523	photosystem II	cellular_component
psbM	GO:0015979	photosynthesis	biological_process
psbM	GO:0016021	integral component of membrane	cellular_component

psbM	GO:0019684	photosynthesis, light reaction	biological_process
psbT	GO:0009523	photosystem II	cellular_component
psbT	GO:0009539	photosystem II reaction center	cellular_component
psbT	GO:0015979	photosynthesis	biological_process
psbT	GO:0016020	membrane	cellular_component
psbZ	GO:0009523	photosystem II	cellular_component
psbZ	GO:0009539	photosystem II reaction center	cellular_component
psbZ	GO:0015979	photosynthesis	biological_process
psbZ	GO:0042549	photosystem II stabilization	biological_process
rbcL	GO:0000287	magnesium ion binding	molecular_function
rbcL	GO:0015977	carbon fixation	biological_process
rbcL	GO:0016984	ribulose-bisphosphate carboxylase activity	molecular_function
rpl14	GO:0003735	structural constituent of ribosome	molecular_function
rpl14	GO:0005840	ribosome	cellular_component
rpl14	GO:0006412	translation	biological_process
rpl14	GO:0015934	large ribosomal subunit	cellular_component
rpl20	GO:0003735	structural constituent of ribosome	molecular_function
rpl20	GO:0005622	intracellular	cellular_component
rpl20	GO:0005840	ribosome	cellular_component
rpl20	GO:0006412	translation	biological_process
rpl20	GO:0019843	rRNA binding	molecular_function
rpl22	GO:0003735	structural constituent of ribosome	molecular_function
rpl22	GO:0005622	intracellular	cellular_component
rpl22	GO:0005840	ribosome	cellular_component
rpl22	GO:0006412	translation	biological_process
rpl22	GO:0015934	large ribosomal subunit	cellular_component
rpl23	GO:0003735	structural constituent of ribosome	molecular_function
rpl23	GO:0005622	intracellular	cellular_component
rpl23	GO:0005840	ribosome	cellular_component
rpl23	GO:0006412	translation	biological_process
rpl2	GO:0003723	RNA binding	molecular_function
rpl2	GO:0003735	structural constituent of ribosome	molecular_function
rpl2	GO:0005840	ribosome	cellular_component
rpl2	GO:0006412	translation	biological_process
rpl2	GO:0015934	large ribosomal subunit	cellular_component
rpl2	GO:0016740	transferase activity	molecular_function
rpl32	GO:0003735	structural constituent of ribosome	molecular_function
rpl32	GO:0006412	translation	biological_process
rpl32	GO:0015934	large ribosomal subunit	cellular_component
rpl33	GO:0003735	structural constituent of ribosome	molecular_function
rpl33	GO:0005622	intracellular	cellular_component

rpl33	GO:0005840	ribosome	cellular_component
rpl33	GO:0006412	translation	biological_process
rpl36	GO:0003735	structural constituent of ribosome	molecular_function
rpl36	GO:0005622	intracellular	cellular_component
rpl36	GO:0005840	ribosome	cellular_component
rpl36	GO:0006412	translation	biological_process
rpoA	GO:0003677	DNA binding	molecular_function
rpoA	GO:0003899	DNA-directed 5'-3' RNA polymerase activity	molecular_function
rpoA	GO:0006351	transcription, DNA-templated	biological_process
rpoA	GO:0046983	protein dimerization activity	molecular_function
rpoB	GO:0003677	DNA binding	molecular_function
rpoB	GO:0003899	DNA-directed 5'-3' RNA polymerase activity	molecular_function
rpoB	GO:0006351	transcription, DNA-templated	biological_process
rpoB	GO:0032549	ribonucleoside binding	molecular_function
rpoC1	GO:0003677	DNA binding	molecular_function
rpoC1	GO:0003899	DNA-directed 5'-3' RNA polymerase activity	molecular_function
rpoC1	GO:0006351	transcription, DNA-templated	biological_process
rpoC2	GO:0003677	DNA binding	molecular_function
rpoC2	GO:0003899	DNA-directed 5'-3' RNA polymerase activity	molecular_function
rpoC2	GO:0006351	transcription, DNA-templated	biological_process
rps11	GO:0003735	structural constituent of ribosome	molecular_function
rps11	GO:0005622	intracellular	cellular_component
rps11	GO:0005840	ribosome	cellular_component
rps11	GO:0006412	translation	biological_process
rps14	GO:0003735	structural constituent of ribosome	molecular_function
rps14	GO:0005622	intracellular	cellular_component
rps14	GO:0005840	ribosome	cellular_component
rps14	GO:0006412	translation	biological_process
rps15	GO:0003735	structural constituent of ribosome	molecular_function
rps15	GO:0005622	intracellular	cellular_component
rps15	GO:0005840	ribosome	cellular_component
rps15	GO:0006412	translation	biological_process
rps16	GO:0003735	structural constituent of ribosome	molecular_function
rps16	GO:0005622	intracellular	cellular_component
rps16	GO:0005840	ribosome	cellular_component
rps16	GO:0006412	translation	biological_process
rps18	GO:0003735	structural constituent of ribosome	molecular_function
rps18	GO:0005622	intracellular	cellular_component
rps18	GO:0005840	ribosome	cellular_component
rps18	GO:0006412	translation	biological_process
rps19	GO:0003723	RNA binding	molecular_function

rps19	GO:0003735	structural constituent of ribosome	molecular_function
rps19	GO:0005840	ribosome	cellular_component
rps19	GO:0006412	translation	biological_process
rps19	GO:0015935	small ribosomal subunit	cellular_component
rps2	GO:0003735	structural constituent of ribosome	molecular_function
rps2	GO:0005622	intracellular	cellular_component
rps2	GO:0005840	ribosome	cellular_component
rps2	GO:0006412	translation	biological_process
rps2	GO:0015935	small ribosomal subunit	cellular_component
rps3	GO:0003723	RNA binding	molecular_function
rps3	GO:0003735	structural constituent of ribosome	molecular_function
rps3	GO:0005840	ribosome	cellular_component
rps3	GO:0006412	translation	biological_process
rps3	GO:0015935	small ribosomal subunit	cellular_component
rps4	GO:0003723	RNA binding	molecular_function
rps4	GO:0003735	structural constituent of ribosome	molecular_function
rps4	GO:0005622	intracellular	cellular_component
rps4	GO:0006412	translation	biological_process
rps4	GO:0015935	small ribosomal subunit	cellular_component
rps4	GO:0019843	rRNA binding	molecular_function
rps7	GO:0003723	RNA binding	molecular_function
rps7	GO:0003735	structural constituent of ribosome	molecular_function
rps7	GO:0006412	translation	biological_process
rps7	GO:0015935	small ribosomal subunit	cellular_component
rps8	GO:0003735	structural constituent of ribosome	molecular_function
rps8	GO:0005840	ribosome	cellular_component
rps8	GO:0006412	translation	biological_process
ycf1	GO:0016021	integral component of membrane	cellular_component
ndhA_2	GO:0016020	membrane	cellular_component
ndhA_2	GO:0055114	oxidation-reduction process	biological_process
ndhB_2	GO:0008137	NADH dehydrogenase (ubiquinone) activity	molecular_function
ndhB_2	GO:0042773	ATP synthesis coupled electron transport	biological_process
ndhB_2	GO:0055114	oxidation-reduction process	biological_process
ndhD_2	GO:0008137	NADH dehydrogenase (ubiquinone) activity	molecular_function
ndhD_2	GO:0016655	oxidoreductase activity, acting on NAD(P)H, quinone or similar compound as acceptor	molecular_function
ndhD_2	GO:0042773	ATP synthesis coupled electron transport	biological_process
ndhD_2	GO:0055114	oxidation-reduction process	biological_process
ndhE_2	GO:0016651	oxidoreductase activity, acting on NAD(P)H	molecular_function
ndhE_2	GO:0042773	ATP synthesis coupled electron transport	biological_process
ndhE_2	GO:0055114	oxidation-reduction process	biological_process

ndhG_2	GO:0008137	NADH dehydrogenase (ubiquinone) activity	molecular_function
ndhG_2	GO:0055114	oxidation-reduction process	biological_process
ndhH_2	GO:0016651	oxidoreductase activity, acting on NAD(P)H	molecular_function
ndhH_2	GO:0048038	quinone binding	molecular_function
ndhH_2	GO:0051287	NAD binding	molecular_function
ndhH_2	GO:0055114	oxidation-reduction process	biological_process
ndhI_2	GO:0008137	NADH dehydrogenase (ubiquinone) activity	molecular_function
ndhI_2	GO:0016020	membrane	cellular_component
ndhI_2	GO:0016651	oxidoreductase activity, acting on NAD(P)H	molecular_function
ndhI_2	GO:0051539	4 iron, 4 sulfur cluster binding	molecular_function
ndhI_2	GO:0055114	oxidation-reduction process	biological_process
psaC_2	GO:0009055	electron transfer activity	molecular_function
psaC_2	GO:0009522	photosystem I	cellular_component
psaC_2	GO:0009773	photosynthetic electron transport in photosystem I	biological_process
psaC_2	GO:0015979	photosynthesis	biological_process
psaC_2	GO:0042651	thylakoid membrane	cellular_component
psaC_2	GO:0051539	4 iron, 4 sulfur cluster binding	molecular_function
rpl23_2	GO:0003735	structural constituent of ribosome	molecular_function
rpl23_2	GO:0005622	intracellular	cellular_component
rpl23_2	GO:0005840	ribosome	cellular_component
rpl23_2	GO:0006412	translation	biological_process
rps15_2	GO:0003735	structural constituent of ribosome	molecular_function
rps15_2	GO:0005622	intracellular	cellular_component
rps15_2	GO:0005840	ribosome	cellular_component
rps15_2	GO:0006412	translation	biological_process
rps7_2	GO:0003723	RNA binding	molecular_function
rps7_2	GO:0003735	structural constituent of ribosome	molecular_function
rps7_2	GO:0006412	translation	biological_process
rps7_2	GO:0015935	small ribosomal subunit	cellular_component
ycf1_2	GO:0016021	integral component of membrane	cellular_component

Supplementary Table S4

The KEGG pathways annotation of genes in *P. asiatica* chloroplast genome			
**Pathway**	**Gene number**	**Gene_list**	**KO_list**
ko01002 Peptidases and inhibitors	1	*clpP1;*	K01358;
ko00640 Propanoate metabolism	1	*accD;*	K01963;
ko00620 Pyruvate metabolism	1	*accD;*	K01963;
ko00710 Carbon fixation in photosynthetic organisms	1	*rbcL;*	K01601;
ko00195 Photosynthesis	32	*atpA;atpB;atpE;atpH;atpI;cemA;pafII;petA;petG;petN;psaA;psaB;psaC_2;psaC;psaI;psaJ;psbA;psbB;psbC;psbD;psbE;psbF;psbH;psbI;psbJ;psbK;psbL;psbM;psbT;psbZ;ycf1_2;ycf1;*	K02111;K02112;K02114;K02110;K02108;K02634;K02634;K02634;K02640;K03689;K02689;K02690;K02691;K02691;K02696;K02697;K02703;K02704;K02705;K02706;K02707;K02708;K02709;K02710;K02711;K02712;K02713;K02714;K02718;K02724;K02690;K02690;
ko00230 Purine metabolism	4	*rpoA;rpoB;rpoC1;rpoC2;*	K03040;K03043;K03046;K03046;
ko00240 Pyrimidine metabolism	4	*rpoA;rpoB;rpoC1;rpoC2;*	K03040;K03043;K03046;K03046;
ko00061 Fatty acid biosynthesis	1	*accD;*	K01963;
ko01212 Fatty acid metabolism	1	*accD;*	K01963;
ko00190 Oxidative phosphorylation	22	*atpA;atpB;atpE;atpH;atpI;ndhA_2;ndhA;ndhB_2;ndhB;ndhC;ndhD_2;ndhD;ndhE_2;ndhE;ndhG_2;ndhG;ndhH_2;ndhH;ndhI_2;ndhI;ndhJ;ndhK;*	K02111;K02112;K02114;K02110;K02108;K05572;K05572;K05573;K05573;K05574;K05575;K05575;K05576;K05576;K05578;K05578;K05579;K05579;K05580;K05580;K05581;K05582;
ko00720 Carbon fixation pathways in prokaryotes	1	*accD;*	K01963;

ko03010 Ribosome	22	*rpl14;rpl20;rpl22;rpl23_2;rpl23;rpl2;rpl32;rpl33;rpl36;rps11;rps14;rps15_2;rps15;rps16;rps18;rps19;rps2;rps3;rps4;rps7_2;rps7;rps8;*	K02874;K02887;K02890;K02892;K02892;K02886;K02911;K02913;K02919;K02948;K02954;K02956;K02956;K02959;K02963;K02965;K02967;K02982;K02986;K02992;K02992;K02994;
ko01200 Carbon metabolism	2	*accD;rbcL;*	K01963;K01601;
ko03020 RNA polymerase	4	*rpoA;rpoB;rpoC1;rpoC2;*	K03040;K03043;K03046;K03046;
ko00630 Glyoxylate and dicarboxylate metabolism	1	*rbcL;*	K01601;

Supplementary Table S5

The type, number, and presence of SSRs in the Cp genome of *P. asiatica*					
**SSR sequence**	**SSR type**	**SSR sequence length**	**Number**	**SSR Location**	**Region**
$(A)_{10}$	mono-nucleotide	10	6	4399-4408, 6935-6944, 7720-7729, 59721-59721, 64688-64697, 69017-69026	LSC
$(A)_{11}$	mono-nucleotide	11	6	11766-11776, 45821-45831, 61453-61463, 74215-74225, 110250-110260,111432-111442	LSC, IRa
$(A)_{12}$	mono-nucleotide	12	8	15935-15946, 29356-29367, 45976-45987, 58034-58045, 59202 -59213, 92845-92856, 128435-128446, 134878-134889	LSC, IRa,IRb
$(A)_{13}$	mono-nucleotide	13	3	4282-4294, 47255-47267, 106955-106967	LSC, IRa
$(A)_{14}$	mono-nucleotide	14	5	1708-1721, 14042-14055,4559-45608, 67343-67356, 116139-116152	LSC, IRa
$(A)_{15}$	mono-nucleotide	15	2	49418-49432, 164964-164978	LSC,IRb
$(A)_{17}$	mono-nucleotide	17	1	68243-68259	LSC
$(T)_{10}$	mono-nucleotide	10	5	5626-5635, 34570-34579, 63875-63884, 68806-68815, 81137-81146	LSC
$(T)_{11}$	mono-nucleotide	11	7	17961-50930, 50920-50930, 74027-74037, 82573-82583, 122346-122356, 136534-136544, 137716-137726	LSC,SSC, IRb
$(T)_{12}$	mono-nucleotide	12	6	7486-7497, 12802-12813, 77026-77037, 113087-113098, 119530-119541, 155120-155131	LSC, IRa,IRb
$(T)_{13}$	mono-nucleotide	13	1	141009-141021	IRb
$(T)_{14}$	mono-nucleotide	14	2	61807-61820, 131824-131837	LSC， IRb

$(T)_{15}$	mono-nucleotide	15	3	79430-79444, 80614-80628, 82998-83012	LSC, IRa
$(TA)_6$	dinucleotide	12	1	7925-7936	LSC
$(AAG)_4$	trinucleotide	12	1	56518-56529	LSC
$(ATA)_4$	trinucleotide	12	1	64971-64982	LSC
$(ATT)_6$	trinucleotide	18	1	20299-20316	LSC
$(TAC)_4$	trinucleotide	12	1	58484-58495	LSC
$(TAT)_4$	trinucleotide	12	1	47055-47066	LSC
$(TTG)_4$	trinucleotide	12	1	53521-53532	LSC
$(AAAT)_3$	tetranucleotide	12	1	20414-20425	LSC
$(AATA)_3$	tetranucleotide	12	1	20423-20434	IRb
$(AGAT)_3$	tetranucleotide	12	1	157372-157383	IRb
$(CAAT)_3$	tetranucleotide	12	1	13098-13109	LSC
$(GGTT)_3$	tetranucleotide	12	1	13107-13118	LSC
$(TATC)_3$	tetranucleotide	12	1	90592-90603	IRa
$(TATT)_3$	tetranucleotide	12	1	120912-120923	IRa
$(AATAA)_3$	pentanucleotide	15	1	58628-58642	LSC
$(TACAA)_3$	pentanucleotide	15	1	86055-86069	IRa
$(TGTAT)_3$	pentanucleotide	15	1	161903-161917	IRb

Supplementary Table S6

**The long tandem repeats identified in *P. asiatica* chloroplast genome**								
**ID**	**Repeat types**	**Repeat length (bp)**	**Repeat unit 1 start**	**Repeat unit 1 end**	**Region**	**Repeat unit 2 start**	**Repeat unit 2 end**	**Region**
R1	F	659	72480	73138	LSC	91711	92369	IRa
R2	P	659	72480	73138	LSC	155605	156263	IRb
R3	P	281	5892	6172	LSC	106506	106786	IRa
R4	F	281	5892	6172	LSC	141188	141468	IRb
R5	P	261	5912	6172	LSC	106506	106766	IRa
R6	F	261	5912	6172	LSC	141208	141468	IRb
R7	F	165	30313	30477	LSC	85306	85470	IRa
R8	P	165	30313	30477	LSC	162504	162668	IRb
R9	F	166	67910	68075	LSC	85080	85245	IRa
R10	P	166	67910	68075	LSC	162729	162894	IRb
R11	F	81	30488	30568	LSC	85512	85592	IRa
R12	P	81	30488	30568	LSC	162382	162462	IRb
R13	F	63	6014	6076	LSC	85015	85077	IRa
R14	P	63	6014	6076	LSC	162897	162959	IRb
R15	P	63	85015	85077	IRa	106602	106664	IRa
R16	F	63	85015	85077	IRa	141310	141372	IRb
R17	F	63	106602	106664	IRa	162897	162959	IRb
R18	P	63	141310	141372	IRb	162897	162959	IRb
R19	F	68	57012	57079	LSC	57057	57124	LSC
R20	F	64	55720	55783	LSC	55761	55824	LSC
R21	F	63	56937	56999	LSC	57072	57134	LSC
R22	F	62	56952	57013	LSC	57102	57163	LSC
R23	F	53	57027	57079	LSC	57072	57124	LSC
R24	P	49	20313	20361	LSC	70195	70243	LSC
R25	F	52	106774	106825	IRa	106866	106917	IRa
R26	P	52	106774	106825	IRa	141057	141108	IRb
R27	P	52	106866	106917	IRa	141149	141200	IRb
R28	F	52	141057	141108	IRb	141149	141200	IRb
R29	F	58	57087	57144	LSC	57102	57159	LSC
R30	F	48	6029	6076	LSC	85030	85077	IRa
R31	P	48	6029	6076	LSC	162897	162944	IRb
R32	P	48	85030	85077	IRa	106602	106649	IRa
R33	F	48	85030	85077	IRa	141325	141372	IRb
R34	P	48	141325	141372	IRb	162897	162944	IRb
R35	F	57	56933	56989	LSC	57023	57079	LSC
R36	F	47	85593	85639	IRa	85662	85708	IRa

R37	P	47	85593	85639	IRa	162266	162312	IRb
R38	P	47	85662	85708	IRa	162335	162381	IRb
R39	F	47	162266	162312	IRb	162335	162381	IRb
R40	P	49	20370	20418	LSC	70138	70186	LSC
R41	F	48	55736	55783	LSC	55777	55824	LSC
R42	P	48	73165	73212	LSC	73165	73212	LSC
R43	F	41	102044	102084	IRa	116416	116456	IRa
R44	P	41	102044	102084	IRa	131518	131558	IRa
R45	P	41	116416	116456	IRa	145890	145930	IRb
R46	F	41	131518	131558	IRb	145890	145930	IRb
R47	F	41	141068	141108	IRb	141160	141200	IRb
R48	F	40	85238	85277	IRa	85637	85676	IRa
R49	P	40	85238	85277	IRa	162298	162337	IRb

Supplementary Table S7

The calculated parameter values for genes in *P.asiatica* chloroplast													
**title**	**T3s**	**C3s**	**A3s**	**G3s**	**CAI**	**CBI**	**Fop**	**Nc**	**GC3s**	**GC**	**L_sym**	**L_aa**	**Gravy**	**Aromo**
rps12	0.4059	0.1881	0.4563	0.1263	0.132	-0.129	0.328	45.31	0.261	0.433	119	120	-0.678333	0.033333
rps12	0.4059	0.1881	0.4563	0.1263	0.132	-0.129	0.328	45.31	0.261	0.433	119	120	-0.678333	0.033333
psbA	0.5248	0.2475	0.3068	0.0864	0.311	0.198	0.533	41.76	0.285	0.422	330	352	0.338352	0.136364
matK	0.5	0.1683	0.416	0.207	0.161	-0.146	0.324	50.91	0.277	0.353	481	502	-0.156773	0.143426
rps16	0.5455	0	0.4286	0.2143	0.204	0.119	0.467	*****	0.2	0.417	15	16	-1.01875	0
psbK	0.537	0.2222	0.2439	0.1667	0.182	-0.169	0.281	38.98	0.316	0.367	57	60	1.27	0.2
psbI	0.5	0.1875	0.36	0.1739	0.208	0.016	0.4	23.64	0.286	0.38	35	36	0.677778	0.194444
atpA	0.4888	0.1521	0.4149	0.1589	0.202	-0.043	0.389	47.01	0.246	0.406	496	507	-0.048718	0.057199
atpF	0.4196	0.1538	0.4276	0.2824	0.152	-0.113	0.354	50.54	0.326	0.387	181	186	-0.48172	0.05914
atpH	0.5211	0.0845	0.3288	0.1791	0.18	-0.03	0.38	49.97	0.228	0.461	79	81	1.002469	0.049383
atpI	0.4434	0.2217	0.3854	0.1429	0.173	-0.057	0.364	50.55	0.297	0.398	239	247	0.650202	0.129555
rps2	0.4462	0.1744	0.3966	0.2138	0.171	-0.106	0.35	53.3	0.301	0.405	226	238	-0.243277	0.071429
rpoC2	0.4325	0.1703	0.4292	0.22	0.143	-0.16	0.318	52.12	0.297	0.393	1340	1377	-0.293028	0.078431
rpoC1	0.4726	0.1734	0.4288	0.1871	0.152	-0.122	0.336	50.25	0.274	0.394	664	687	-0.295488	0.09607
rpoB	0.4457	0.1412	0.4562	0.2054	0.146	-0.132	0.332	49.97	0.264	0.396	1036	1070	-0.273738	0.082243
petN	0.36	0.2	0.4091	0.0952	0.103	-0.333	0.16	32.76	0.28	0.448	25	29	1.544827	0.137931
psbM	0.5667	0.2	0.3846	0	0.226	-0.086	0.333	42.67	0.182	0.324	33	34	1.429412	0.117647
psbD	0.4749	0.2241	0.3265	0.181	0.24	0.052	0.447	50.02	0.329	0.445	331	353	0.341926	0.169972
psbC	0.4716	0.1753	0.3938	0.1429	0.176	-0.076	0.364	44.24	0.263	0.431	448	473	0.264482	0.141649
psbZ	0.4912	0.1754	0.32	0.1136	0.24	0.043	0.407	49.15	0.254	0.371	59	62	1.416129	0.112903
rps14	0.3733	0.1467	0.494	0.2078	0.126	-0.181	0.302	42.28	0.281	0.403	96	100	-0.98	0.07

psaB	0.4904	0.1821	0.373	0.1627	0.185	-0.096	0.36	49.41	0.276	0.416	688	734	0.129292	0.148501
psaA	0.4579	0.19	0.3766	0.1811	0.207	-0.072	0.373	48.66	0.297	0.433	707	750	0.247867	0.132
ycf3	0.4812	0.1654	0.4274	0.2427	0.153	-0.229	0.311	57.32	0.292	0.397	161	168	-0.522024	0.142857
rps4	0.4591	0.1761	0.439	0.1722	0.164	-0.046	0.377	50.21	0.271	0.392	199	203	-0.600985	0.064039
ndhJ	0.4961	0.1654	0.4107	0.1979	0.143	-0.198	0.295	57.5	0.268	0.397	149	158	-0.298734	0.139241
ndhK	0.4737	0.1632	0.4583	0.1242	0.137	-0.231	0.276	45.93	0.23	0.387	217	225	-0.258222	0.102222
ndhC	0.51	0.1	0.3793	0.2297	0.172	-0.132	0.306	51.95	0.243	0.361	111	120	1.045833	0.208333
atpE	0.4623	0.1038	0.4167	0.2292	0.148	-0.123	0.331	53.61	0.26	0.406	127	133	-0.080451	0.022556
atpB	0.4436	0.1961	0.396	0.1671	0.204	-0.013	0.402	50.24	0.294	0.43	483	498	0.003012	0.062249
rbcL	0.4846	0.2	0.3972	0.1571	0.275	0.1	0.48	47.54	0.283	0.437	460	477	-0.246751	0.098532
accD	0.4504	0.17	0.3639	0.3432	0.177	-0.162	0.327	52.44	0.368	0.389	446	463	-0.43283	0.084233
psaI	0.5484	0.129	0.3846	0.125	0.257	-0.095	0.324	37.29	0.206	0.343	34	36	1.188889	0.138889
ycf4	0.4675	0.1818	0.3714	0.1951	0.156	-0.059	0.369	53.94	0.295	0.4	176	184	0.173913	0.146739
cemA	0.5304	0.1768	0.4136	0.1603	0.194	-0.073	0.366	44.74	0.245	0.319	216	229	0.272489	0.131004
petA	0.4922	0.168	0.358	0.2379	0.202	-0.032	0.39	52.66	0.308	0.408	315	320	-0.084687	0.075
psbJ	0.5526	0.1053	0.303	0.1154	0.16	-0.011	0.395	37.23	0.184	0.375	38	40	1.1475	0.125
psbL	0.4375	0.2813	0.4167	0.1304	0.191	-0.015	0.389	47.54	0.333	0.333	36	38	0.257895	0.184211
psbF	0.3235	0.2353	0.4483	0.16	0.13	-0.154	0.306	52.36	0.333	0.427	36	39	0.738462	0.153846
psbE	0.4932	0.2055	0.4127	0.0556	0.173	-0.054	0.387	50.54	0.225	0.394	80	83	0.046988	0.156627
petL	0.5517	0.069	0.3846	0.0952	0.124	0.031	0.4	29.95	0.133	0.312	30	31	1.66129	0.129032
petG	0.5152	0.0909	0.4138	0.16	0.156	-0.059	0.333	41.8	0.194	0.333	36	37	1.102702	0.135135
psaJ	0.375	0.15	0.4194	0.2759	0.117	-0.251	0.238	47.79	0.333	0.417	42	44	0.847727	0.204545
rpl33	0.36	0.26	0.4468	0.2927	0.176	-0.151	0.344	58.47	0.391	0.379	64	66	-0.725758	0.060606
rps18	0.4688	0.1563	0.4078	0.2421	0.134	-0.048	0.384	40.53	0.304	0.352	125	126	-1.134921	0.063492

rpl20	0.45	0.15	0.5052	0.1687	0.148	-0.043	0.39	43	0.236	0.36	123	126	-0.672222	0.071429
clpP1	0.3567	0.2573	0.4052	0.2031	0.184	-0.055	0.383	58.78	0.363	0.443	193	198	0.284849	0.075758
psbB	0.4862	0.1751	0.3459	0.1895	0.193	-0.043	0.392	48.94	0.294	0.446	480	508	0.109646	0.147638
psbT	0.4444	0.1111	0.5385	0.1364	0.18	-0.043	0.375	*****	0.188	0.303	32	33	0.887879	0.181818
psbN	0.3056	0.3333	0.4545	0.125	0.184	-0.068	0.381	40.73	0.381	0.465	42	43	0.181395	0.139535
psbH	0.4426	0.0984	0.4333	0.1852	0.142	-0.122	0.333	45.61	0.232	0.383	69	74	0.254054	0.067568
rpoA	0.4618	0.1832	0.4781	0.1674	0.175	-0.088	0.362	47.51	0.261	0.357	326	335	-0.31791	0.086567
rps11	0.4118	0.1597	0.4224	0.1509	0.147	-0.122	0.338	52.25	0.263	0.466	133	138	-0.492029	0.036232
rpl36	0.3448	0.2414	0.5161	0.12	0.131	-0.008	0.389	*****	0.278	0.414	36	37	-0.748649	0
infA	0.4262	0.1639	0.4407	0.2449	0.153	-0.097	0.351	54.32	0.297	0.39	74	77	-0.607792	0.077922
rps8	0.4211	0.1579	0.4722	0.1461	0.103	-0.032	0.377	42.78	0.238	0.368	130	134	-0.381343	0.052239
rpl14	0.43	0.2	0.4792	0.1098	0.186	0.032	0.424	44.14	0.246	0.402	118	122	-0.029508	0.040984
rps3	0.4172	0.2025	0.5525	0.1242	0.175	-0.115	0.359	43.72	0.236	0.354	220	225	-0.448	0.102222
rpl22	0.4412	0.1397	0.5224	0.1597	0.186	-0.066	0.381	48.15	0.226	0.36	168	172	-0.462209	0.093023
rps19	0.4932	0.1507	0.5571	0.0508	0.19	-0.103	0.36	39.06	0.157	0.33	89	92	-0.408696	0.065217
rpl2	0.3932	0.2051	0.4136	0.1919	0.134	-0.107	0.353	54.76	0.32	0.44	269	274	-0.610219	0.047445
rpl23	0.4658	0.1233	0.4638	0.1774	0.123	-0.248	0.267	47.15	0.233	0.376	86	93	-0.43871	0.096774
rps12	0.4059	0.1881	0.4563	0.1263	0.132	-0.129	0.328	45.31	0.261	0.433	119	120	-0.678333	0.033333
ycf2	0.4287	0.2047	0.3876	0.2797	0.149	-0.141	0.331	53.95	0.357	0.391	1796	1863	-0.409554	0.117016
rps7	0.4274	0.1368	0.5267	0.1167	0.195	-0.043	0.396	44.8	0.201	0.394	149	155	-0.628387	0.03871
ndhB	0.4343	0.2136	0.4192	0.1218	0.162	-0.088	0.351	48.12	0.276	0.376	467	492	0.680488	0.123984
rps16	0.5455	0	0.4286	0.2143	0.204	0.119	0.467	*****	0.2	0.417	15	16	-1.01875	0
ycf1	0.4651	0.1797	0.4989	0.2037	0.168	-0.094	0.364	50.62	0.273	0.33	1875	1953	-0.562161	0.126984

Supplementary Table S8

The relative synonymous codon usage of the *P. asiatica* chloroplast genome						
**Amino acids**	**Codon**	**Number**	**Ratio of Codon**	**RSCU**	**Number of amino acids**	**Ratio of amino acid**
Leu	UUA	979	3.38%	1.82	3220	11.12%
	CUU	669	2.31%	1.256		
	UUG	663	2.29%	1.24		
	CUA	496	1.71%	0.918		
	CUG	210	0.73%	0.387		
	CUC	203	0.70%	0.379		
Ile	AUU	1156	3.99%	1.467	2362	8.16%
	AUA	716	2.47%	0.921		
	AUC	490	1.69%	0.612		
Ser	UCU	630	2.18%	1.686	2215	7.65%
	AGU	440	1.52%	1.211		
	UCA	412	1.42%	1.11		
	UCC	376	1.30%	1.022		
	UCG	240	0.83%	0.656		
	AGC	117	0.40%	0.315		
Gly	GGA	729	2.52%	1.567	1853	6.40%
	GGU	562	1.94%	1.246		
	GGG	357	1.23%	0.759		
	GGC	205	0.71%	0.427		
Arg	AGA	500	1.73%	1.738	1729	5.97%
	CGA	418	1.44%	1.431		
	CGU	366	1.26%	1.284		
	AGG	213	0.74%	0.738		
	CGG	128	0.44%	0.444		
	CGC	104	0.36%	0.365		
Thr	ACU	556	1.92%	1.548	1666	5.75%
	ACC	415	1.43%	0.779		
	ACA	415	1.43%	1.174		
	ACG	280	0.97%	0.499		
Phe	UUU	1081	3.73%	1.312	1658	5.73%
	UUC	577	1.99%	0.688		
Lys	AAA	1224	4.23%	1.496	1643	5.68%
	AAG	419	1.45%	0.504		
Glu	GAA	1167	4.03%	1.493	1570	5.42%
	GAG	403	1.39%	0.507		
Ala	GCU	658	2.27%	1.693	1531	5.29%
	GCA	428	1.48%	1.141		

	GCC	274	0.95%	0.706		
	GCG	171	0.59%	0.46		
Val	GUU	576	1.99%	1.527	1516	5.24%
	GUA	538	1.86%	1.412		
	GUC	201	0.69%	0.519		
	GUG	201	0.69%	0.542		
Asn	AAU	1026	3.54%	1.564	1314	4.54%
	AAC	288	0.99%	0.436		
Pro	CCU	426	1.47%	1.457	1206	4.17%
	CCA	350	1.21%	1.138		
	CCC	269	0.93%	0.864		
	CCG	161	0.56%	0.541		
Asp	GAU	944	3.26%	1.582	1187	4.10%
	GAC	243	0.84%	0.418		
Tyr	UAU	806	2.78%	1.592	1013	3.50%
	UAC	207	0.72%	0.408		
Gln	CAA	777	2.68%	1.545	1009	3.49%
	CAG	232	0.80%	0.455		
His	CAU	503	1.74%	1.536	660	2.28%
	CAC	157	0.54%	0.464		
Met	AUG	644	2.22%	1	644	2.22%
Trp	UGG	508	1.75%	1	508	1.75%
Cys	UGU	267	0.92%	1.501	352	1.22%
	UGC	85	0.29%	0.499		
TER	UAA	48	0.17%	1.548	70	0.24%
	UGA	23	0.08%	0.742		
	UAG	22	0.08%	0.710		
RSCU, Relative synonymous codon usage						

Supplementary Table S9

**Gene Pi analysis of *P. asiatica***	
Gene	Pi
ndhB	0.02263
psbL	0.02637
rps12	0.02901
rps7	0.0294
petN	0.0366
psbA	0.03786
rpl2	0.03874
psbE	0.04308
psaI	0.04375
ycf3	0.04448
psbD	0.0456
psaA	0.0479
psbJ	0.04814
atpH	0.04923
psbF	0.05142
petG	0.05154
psaB	0.05269
psbC	0.05355
petD	0.05555
psbM	0.05758
psaC	0.05781
petB	0.05856
psbT	0.05934
ndhC	0.06104
petL	0.06386
psaJ	0.06774
psbI	0.06777
rbcL	0.06859
rpoC1	0.0692
ndhJ	0.06958
rpoB	0.07242
ndhK	0.07245
atpB	0.07368
atpI	0.07473
atpA	0.07687
ndhH	0.07905
ndhE	0.07986
ndhI	0.08109

rpl14	0.08114
rps2	0.08138
petA	0.08417
rps4	0.08422
atpE	0.08449
rpl36	0.0848
ndhA	0.08679
ycf2	0.08769
ndhG	0.08832
psbK	0.09009
ycf4	0.09325
rps18	0.09493
ndhD	0.09522
rpoA	0.09588
psbH	0.09634
rpoC2	0.10784
rps3	0.10826
accD	0.11221
rpl32	0.1145
rps11	0.11529
ycf1	0.11604
rpl20	0.1165
rps8	0.11752
ccsA	0.12551
rpl33	0.12724
rps15	0.13182
ndhF	0.14189
rpl16	0.14646
rpl22	0.14854
matK	0.1536
clpP	0.23005
